# Colossal Strain Tuning of Ferroelectric Transitions in KNbO_3_ Thin Films

**DOI:** 10.1002/adma.202408664

**Published:** 2024-11-12

**Authors:** Sankalpa Hazra, Tobias Schwaigert, Aiden Ross, Haidong Lu, Utkarsh Saha, Victor Trinquet, Betul Akkopru‐Akgun, Benjamin Z. Gregory, Anudeep Mangu, Suchismita Sarker, Tatiana Kuznetsova, Saugata Sarker, Xin Li, Matthew R. Barone, Xiaoshan Xu, John W. Freeland, Roman Engel‐Herbert, Aaron M. Lindenberg, Andrej Singer, Susan Trolier‐McKinstry, David A. Muller, Gian‐Marco Rignanese, Salva Salmani‐Rezaie, Vladimir A. Stoica, Alexei Gruverman, Long‐Qing Chen, Darrell G. Schlom, Venkatraman Gopalan

**Affiliations:** ^1^ Department of Materials Science and Engineering Pennsylvania State University University Park PA 16802 USA; ^2^ Platform for the Accelerated Realization, Analysis, and Discovery of Interface Materials (PARADIM) Cornell University Ithaca NY 14853 USA; ^3^ Department of Materials Science and Engineering Cornell University Ithaca NY 14853 USA; ^4^ Department of Physics and Astronomy University of Nebraska Lincoln NE 68588 USA; ^5^ Institute of Condensed Matter and Nanosciences UCLouvain Louvain‐la‐Neuve 1348 Belgium; ^6^ Department of Physics Cornell University Ithaca NY 14853 USA; ^7^ Department of Materials Science and Engineering Stanford University Stanford CA 94305 USA; ^8^ Stanford Institute for Materials Energy Sciences SLAC National Accelerator Laboratory Menlo Park CA 94025 USA; ^9^ Cornell High Energy Synchrotron Source Cornell University Ithaca NY 14853 USA; ^10^ Advanced Photon Source Argonne National Laboratory Argonne IL 60439 USA; ^11^ Paul‐Drude‐Institut für Festkörperelektronik Leibniz‐Institut im Forschungsverbund Berlin e.V. Hausvogteiplatz 5 10117 Berlin Germany; ^12^ School of Applied and Engineering Physics Cornell University Ithaca NY 14853 USA; ^13^ Department of Materials Science and Engineering Ohio State University Columbus OH 43210 USA; ^14^ Kavli Institute at Cornell for Nanoscale Science Ithaca NY 14853 USA; ^15^ Leibniz‐Institut für Kristallzüchtung Max‐Born‐Straße 2 12489 Berlin Germany

**Keywords:** ferroelectrics, phase‐field modeling, second harmonic generation, strain‐tuning, thin films

## Abstract

Strong coupling between polarization (*P*) and strain (ɛ) in ferroelectric complex oxides offers unique opportunities to dramatically tune their properties. Here colossal strain tuning of ferroelectricity in epitaxial KNbO_3_ thin films grown by sub‐oxide molecular beam epitaxy is demonstrated. While bulk KNbO_3_ exhibits three ferroelectric transitions and a Curie temperature (*T_c_*) of ≈676 K, phase‐field modeling predicts that a biaxial strain of as little as −0.6% pushes its *T_c_* > 975 K, its decomposition temperature in air, and for −1.4% strain, to *T_c_* > 1325 K, its melting point. Furthermore, a strain of −1.5% can stabilize a single phase throughout the entire temperature range of its stability. A combination of temperature‐dependent second harmonic generation measurements, synchrotron‐based X‐ray reciprocal space mapping, ferroelectric measurements, and transmission electron microscopy reveal a single tetragonal phase from 10 K to 975 K, an enhancement of ≈46% in the tetragonal phase remanent polarization (*P_r_*), and a ≈200% enhancement in its optical second harmonic generation coefficients over bulk values. These properties in a lead‐free system, but with properties comparable or superior to lead‐based systems, make it an attractive candidate for applications ranging from high‐temperature ferroelectric memory to cryogenic temperature quantum computing.

## Graphical abstract

Colossal strain tuning of ferroelectricity is demonstrated in biaxially compressive strained epitaxial KNbO_3_ thin films, demonstrating a dramatic strain enhancement of ferroelectric polarization and the Curie temperature, eliminating all bulk phase transitions and stabilizing a single tetragonal phase from 5 K to 975 K.

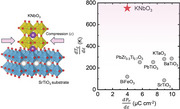

## 1 Introduction

The ability to apply large strains in epitaxial thin films makes it possible to engineer emergent phases in materials with enhanced material properties that are inaccessible in their bulk form.^[^ ^1^ ^]^ Owing to a strong coupling between strain and polar order in ferroelectrics, large shifts in properties such as paraelectric‐to‐ferroelectric transition temperature (*T_c_*) and remanent polarization (*P_r_*) have been theoretically predicted and experimentally observed.^[^ ^2^, ^3^, ^4^, ^5^, ^6^ ^]^ Notable examples of strain‐enhanced properties and tunable phase transitions in perovskite oxides include a large shift in *T_c_* in BaTiO_3_,^[^ ^2^ ^]^ observation of room temperature ferroelectricity in quantum paraelectric SrTiO_3_,^[^ ^3^, ^4^ ^]^ and the realization of super‐tetragonal BiFeO_3_.^[^ ^7^ ^]^ Given that there are practical limits to epitaxially straining a ceramic thin film on a rigid single crystal substrate to typically a few percent (a maximum of 6.6% has been demonstrated^[^ ^7^ ^]^), one ideally desires the maximum strain *tunability* of the film properties, measured in units of property per unit strain, such as $\frac{dT_{c}}{d\varepsilon }$ and $\frac{dP_{r}}{d\varepsilon }$.

Moreover, to achieve the desired phase over a broad temperature range, undesirable thermal phase transitions require suppression. For instance, in BaTiO_3_‐based electrooptic modulators employed in quantum computing at cryogenic temperatures, symmetry‐lowering phase transitions in BaTiO_3_ significantly diminish the superior electrooptic properties of the room‐temperature tetragonal phase.^[^ ^8^ ^]^ Stabilization of the tetragonal phase in this system at cryogenic operational temperatures would thus be ideal. For high‐temperature ferroelectric memory and actuation applications, the stabilization of ferroelectricity at high temperatures through a large *T_c_* is desired. Further, environmentally benign ferroelectrics^[^ ^9^, ^10^ ^]^ are desired, in contrast to Pb‐based ferroelectrics.

Strain tuning of environmentally benign perovskite alkali niobates have been explored due to their strong ferroelectricity,^[^ ^11^ ^]^ piezoelectricity,^[^ ^12^, ^13^ ^]^ and nonlinear optical responses.^[^ ^14^ ^]^ In (Na,K)NbO_3_ films, strain tuning proves to be an effective pathway to control phase transitions,^[^ ^15^, ^16^ ^]^ while allowing properties competing with those of Pb‐based ferroelectrics.^[^ ^17^ ^]^ Unfortunately, NaNbO_3_ is highly polymorphic as compared with KNbO_3_, making pure KNbO_3_ a more suitable material system to establish a single structural phase across a large temperature range.^[^ ^18^ ^]^ While strain‐relaxed KNbO_3_ films have been studied,^[^ ^19^, ^20^, ^21^, ^22^, ^23^, ^24^ ^]^ strain tuning of this Pb‐free, environmentally benign system has not been explored thus far. Furthermore, prior demonstrations of the growth of KNbO_3_ on a silicon substrate^[^ ^25^, ^26^ ^]^ make KNbO_3_ appealing for semiconductor device integration.

In this work, we demonstrate that strain‐tuning of KNbO_3_ provides a colossal strain tunability of its *T_c_*, while retaining the desirable tetragonal phase from 10 K to 975 K, its decomposition temperature in air. Strain mediates the stabilization of a superior tetragonal phase in KNbO_3_, mimicking the tetragonality of PbTiO_3_ but with much higher *T_c_*, and significantly larger nonlinear optical coefficients.

## 2 Theory Prediction of the Strain Phase Diagram

Bulk KNbO_3_ has a rich phase diagram undergoing a series of phase transitions from cubic to tetragonal at 676 K, tetragonal to orthorhombic at 492 K, and eventually orthorhombic to rhombohedral at 223 K.^[^ ^11^ ^]^ To predict the influence of biaxial strain on the lattice parameters and temperature‐dependent phase diagram of KNbO_3_, we performed phase‐field simulations of biaxially compressed KNbO_3_ thin films.

**Figure 1:**
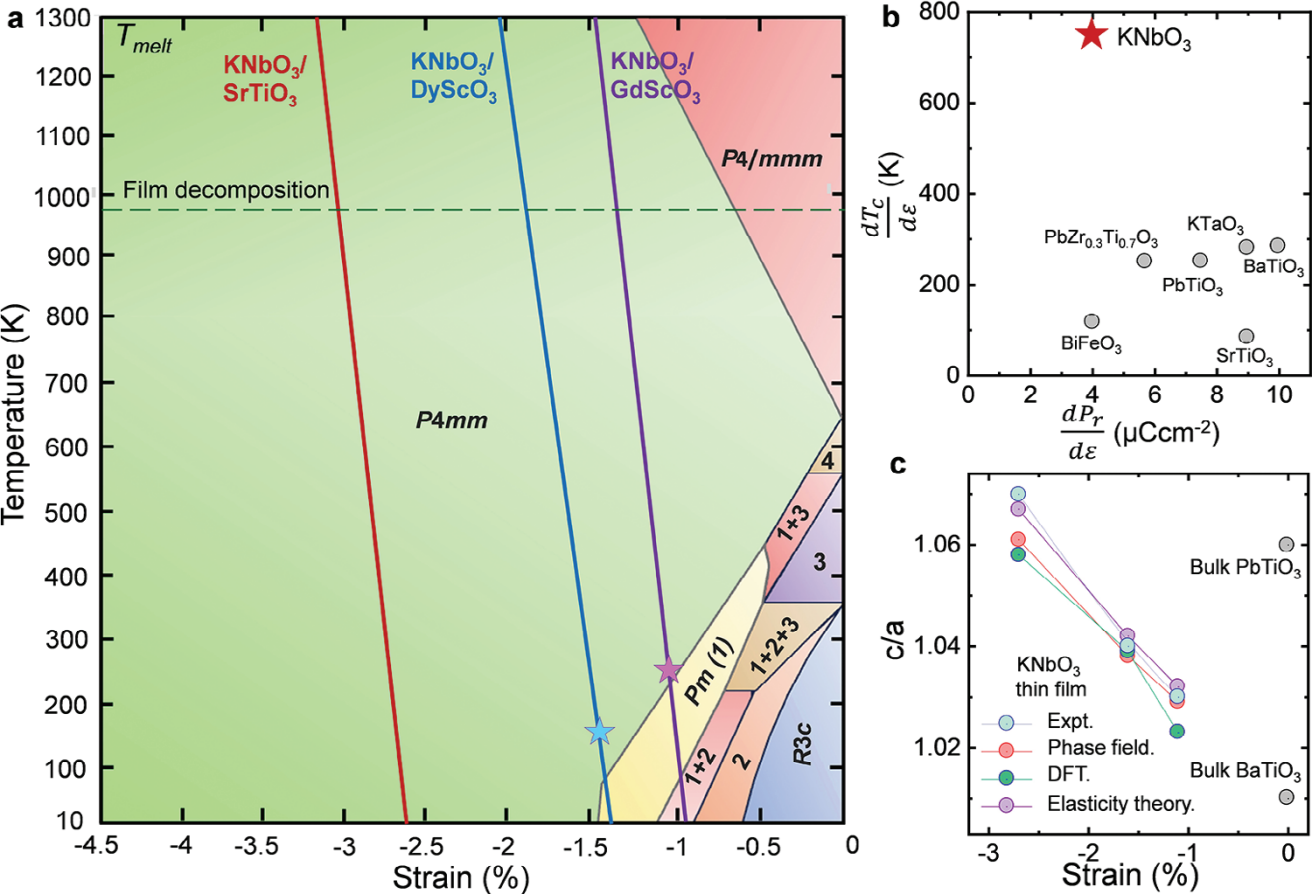
a) Thermodynamic phase‐field simulations of biaxially compressed KNbO_3_. The three different strain films on SrTiO_3_, DyScO_3_, and GdScO_3_ studied here are marked by red, blue, and violet lines. Legend for numerically labeled phases: 1: *Pm*, 2: *Cm*, 3: *Amm*2, 4: Phase mixture of *P*4*mm* with *c*‐axis poled in the in‐plane direction and out‐of‐plane direction. Blue and violet stars indicate the onset of the tetragonal‐to‐monoclinic transition temperature observed experimentally. b) $\frac{dT_{c}}{d\varepsilon }$ and $\frac{dP_{r}}{d\varepsilon }$ plotted for KNbO_3_ in comparison to related materials: BaTiO_3_,^[^ ^27^, ^75^ ^]^ PbTiO_3_,^[^ ^1^, ^27^, ^76^ ^]^ PbZr_0.3_Ti_0.7_O_3_,^[^ ^28^, ^77^ ^]^ SrTiO_3_,^[^ ^3^, ^78^ ^]^ KTaO_3_,^[^ ^79^, ^80^ ^]^ and BiFeO_3_.^[^ ^7^, ^27^, ^81^ ^]^ $\frac{dT_{c}}{d\varepsilon }$ for KNbO_3_ is labeled by predicted values from phase‐field simulations. c) *c/a* ratio (out‐of‐plane to in‐plane lattice parameter ratio) plotted as a function of strain experimentally measured for KNbO_3_ thin films at room temperature compared to phase‐field predictions, DFT calculations, and elasticity theory calculations (see Supporting information), showing the effect of strain in enhancing the stability of the tetragonal phase in KNbO_3_.

**Figure** **1**a depicts the phase diagram predicted from phase‐field simulations as a function of biaxial epitaxial strain, *ɛ* = (*a*_||_−*a*_o_)/*a*_o_, where *a*_o_ is the effective cubic lattice parameter extrapolated from the high‐temperature KNbO_3_ cubic phase and *a*_||_ is the in‐plane lattice parameter of the biaxially strained KNbO_3_. Under a compressive strain of ≈ −0.7%, it is possible to stabilize the high‐temperature tetragonal phase at room temperature where the orthorhombic phase is observed in its bulk counterpart. Further, a strain of ≈ −1.5% is sufficient to stabilize the tetragonal phase down to 0 K, eliminating all other phase transitions. At such strains, the Curie temperature, *T_c_* is predicted to be enhanced beyond its melting temperature of 1325 K^[^ ^18^ ^]^ as seen in Figure 1a. Strikingly, the rate of change of *T_c_* with respect to strain, i.e., $\frac{dT_{c}}{d\varepsilon }$, is predicted to be a factor of ≈3  × higher compared to most well‐known ferroelectrics as shown in Figure 1b, while its rate of change of remanent polarization with respect to strain, $\frac{dP_{r}}{d\varepsilon }$, is predicted to be intermediate between that of tetragonal BiFeO_3_^[^ ^27^ ^]^ and PbZr_0.3_Ti_0.7_O_3_.^[^ ^28^ ^]^

The superior stability of the tetragonal phase under higher compressive strains is driven by its better match with strain conditions imposed by the substrate, resulting in minimal elastic energy in comparison to other possible phases. For lower compressive strains, KNbO_3_ is predicted to undergo a transition from the tetragonal to a lower symmetry monoclinic phase instead of an orthorhombic phase as seen in bulk KNbO_3_. This is because of the large enhancement of the *c/a* lattice parameter ratio (Figure 1c) due to the epitaxial clamping to the substrate, effectively distorting the orthorhombic phase in bulk KNbO_3_ into a monoclinic phase under compressive strain (see Supporting Information). At room temperature, the *c/a* ratio increases from its bulk value of ≈1.01 (similar to that of bulk BaTiO_3_) to a value of ≈1.07 (larger than that for bulk PbTiO_3_) at −2.7% strain. The polarization direction and lattice structure of the strain‐stabilized tetragonal and monoclinic phases in the KNbO_3_ thin films are shown in Figure S1 (Supporting Information).

Density functional theory calculations also predict the stability of the monoclinic phase to be comparable to the tetragonal phase for intermediate strains (Figure S2, Supporting Information). From finite‐temperature phase‐field simulations, it is evident that for higher compressive strains (more than ≈ −1.5%) all symmetry‐lowering phase transitions can be eliminated and the single desirable tetragonal phase can be stabilized across the entire temperature regime from 0 K to *T_c_*.

From thermodynamic calculations, the Curie temperature, *T_c_*, for the tetragonal *c*‐phase under a biaxial compressive strain (*c*‐axis perpendicular to the substrate) is given by,

(1) $$T_{c}\left(\varepsilon \right)=T_{c}\left(\varepsilon =0\right)+4\varepsilon_{o}C\left(\frac{Q_{13}}{S_{11}+S_{13}}\right)\left|\varepsilon \right|$$

where *C* is the Curie–Weiss constant, *Q* is the electrostrictive coefficient, *S* is the elastic compliance, and ɛ_o_ is the vacuum permittivity. Comparing the *C*, *Q*, and *S* of KNbO_3_ versus those for PbTiO_3_, BaTiO_3,_ and SrTiO_3_, (Table S3 Supporting Information) it is evident that the strong sensitivity of *T_c_* to strain is predominantly due to a larger electrostrictive coefficient, *Q*_13_, in KNbO_3_. For example, *C*, $\frac{1}{S_{11}+S_{13}}$, and *Q*_13_ are ≈6%, ≈40%, and ≈200% larger in KNbO_3_ relative to PbTiO_3_, thus leading to a ≈3  × larger $\frac{dT_{c}}{d\varepsilon }$.

Following Equation (1), it is evident that a larger *Q*_13_ and lower *S*_11_ + *S*_13_ translates to a higher *T_c_* and $\frac{dT_{c}}{d\varepsilon }$. Unfortunately, this linear relationship does not extend to the case of *P_r_* and $\frac{dP_{r}}{d\varepsilon }$, where there are optimal values for *Q* and *S* which maximize *P_r_* and $\frac{dP_{r}}{d\varepsilon }$, as shown in Figure S3 (Supporting Information). In particular, the *Q*_13_ and *S*_11_ + *S*_13_ values of KNbO_3_ do not optimize the maximum possible value of *P_r_* and $\frac{dP_{r}}{d\varepsilon };$ nonetheless these values are highly competitive with other material systems as shown in Figure 1b.

## 3 Epitaxy and Phase Transitions by Optical Second Harmonic Generation

To experimentally measure the strain phase diagram, single crystal (001)_c_ SrTiO_3_, (110)_o_ DyScO_3_, and (110)_o_ GdScO_3_ (subscript “c” for cubic, “o” for orthorhombic) were used as compatible substrates, which allows us to impart compressive strain, *ɛ*, of ≈−2.7%, −1.6%, and −1.1%, respectively, on (001) KNbO_3_ films. (To be precise, the biaxial in‐plane strain for the scandate substrates is anisotropic, namely −1.65% and −1.5% for DyScO_3_, and −1.15% and −1.05% for GdScO_3_. Note that this difference was not experimentally resolved and is hence ignored in the rest of this study). Epitaxial KNbO_3_ thin films were grown by suboxide molecular‐beam epitaxy with in situ reflection high‐energy electron diffraction (RHEED) (Figure S4, Supporting information). Atomic force microscopy (AFM) was used to confirm smooth surface morphology with root‐mean‐square (rms) roughness < 1 nm for all three films (Figure S5, Supporting information). X‐ray reciprocal space mapping (RSM) confirms the commensurately strained nature of the thin films on all three substrates (Figures S6 and S7, Supporting information). Cross‐sectional transmission electron microscopy shows a uniform interface structure with minimum interface intermixing, with the film being coherently strained to the substrate with no extended defects (Figures S8–S11 Supporting information). X‐ray photoemission spectroscopy confirms the expected valence states of K, Nb, and O in KNbO_3_ films (Figure S12, Supporting Information).

**Figure 2:**
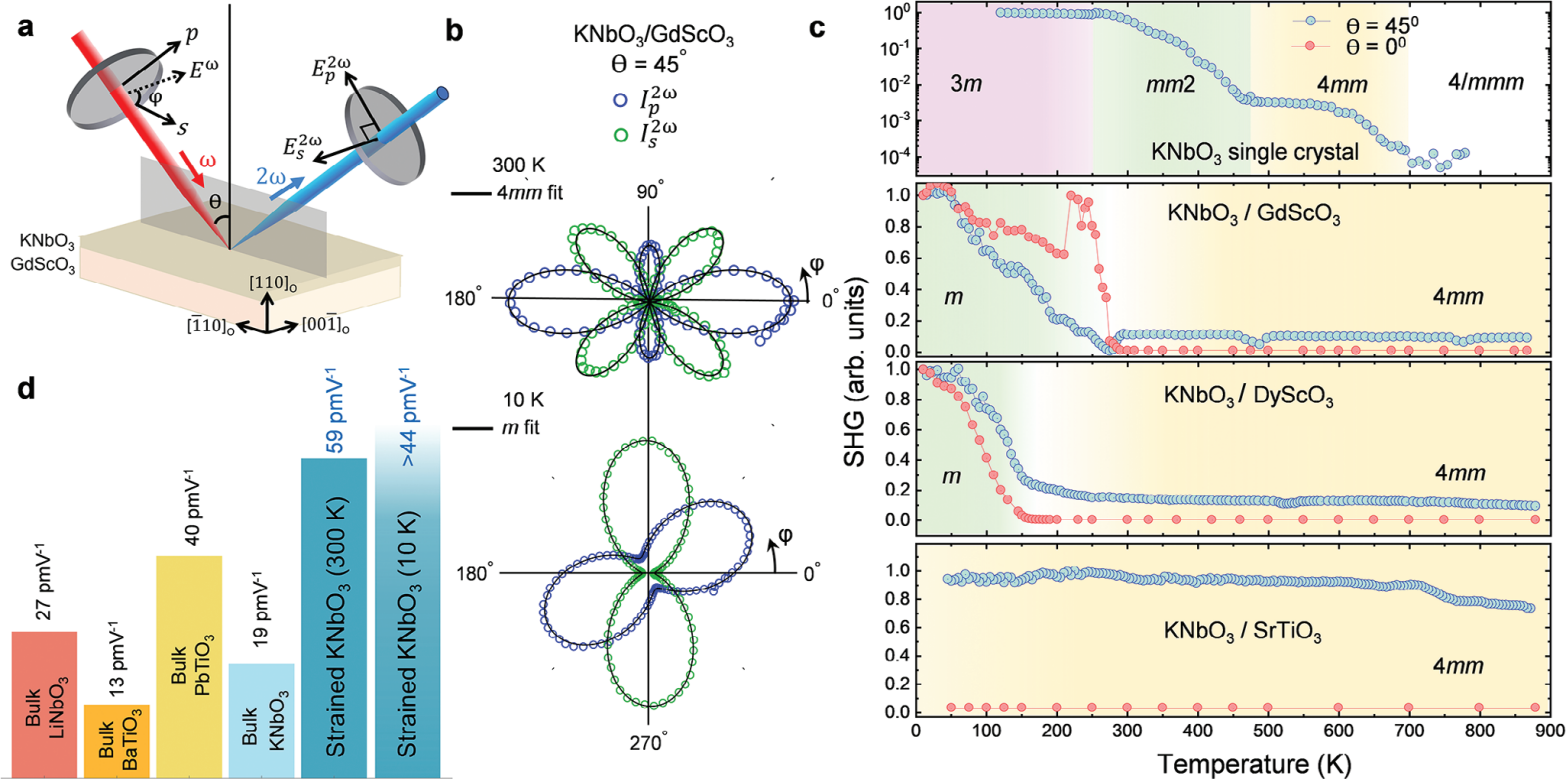
a) Schematic of second harmonic generation (SHG) setup in reflection geometry. b) SHG polar plots for KNbO_3_ on GdScO_3_ at 300 K (upper panel) and 10 K (lower panel) fitted to tetragonal and multidomain monoclinic models, respectively, showing symmetry lowering from room temperature to low temperature consistent with phase‐field predictions. Equations for SHG modeling are discussed in the Supporting information. c) Normal and oblique‐incidence SHG intensity versus temperature between 10 K and 900 K shown for bulk KNbO_3_ single crystal, and strained KNbO_3_ films on GdScO_3_, DyScO_3_, and SrTiO_3_ substrates. Bulk phase transition temperatures are consistent with previous reports. Normal‐incidence SHG signal (red traces) shows signatures of transition from tetragonal to a lower symmetry monoclinic phase since it is sensitive only to the latter phase. With increasing compressive strain, this symmetry‐lowering phase transition (marked by green regions) is pushed down in temperature and eventually eliminated in the highest strained KNbO_3_ on SrTiO_3_. d) Nonlinear optical coefficients (*d*_33_) calculated from SHG modeling for KNbO_3_ thin films for both 300 K and 10 K compared to bulk room temperature values in similar materials. Due to the multidomain model for the low temperature monoclinic phase, only a lower bound for the *d*_11_ monoclinic coefficient could be extracted.

To experimentally identify the phase diagram of the strained KNbO_3_ thin films, temperature‐dependent second harmonic generation (SHG) measurements were performed. Electric‐dipole SHG is a nonlinear optical process where incident light at the fundamental frequency of ω is converted into the SHG frequency of 2ω due to the broken inversion symmetry in the material.^[^ ^29^ ^]^ A schematic of the setup used for SHG measurements is shown in **Figure** **2**a. By rotating the incident polarization of light at the fundamental frequency, SHG polar plots can be measured corresponding to two orthogonally polarized (*s* and *p*‐polarized) second harmonic light reflected from the sample surface; this is called SHG polarimetry. By fitting the polar plots, the point group symmetry of the thin films can be determined.

Figure 2b shows the oblique incidence SHG polarimetry for the KNbO_3_ on GdScO_3_ film (lowest biaxial strain, *ɛ* ≈ −1.1%) at 300 K fitted to a 4*mm* (tetragonal) model (see Supporting information), validating the influence of the strain in stabilizing a tetragonal phase of KNbO_3_ at room temperature even for the lowest strained thin film of the set. A tetragonal unit cell with polarization completely out‐of‐plane will exhibit no SHG signal at normal incidence. Nonetheless, symmetry lowering from a tetragonal to a lower symmetry phase will generate SHG at normal incidence due to the appearance of an in‐plane polarization component in lower symmetry phases. Temperature‐dependent SHG at normal incidence indeed shows that KNbO_3_ on GdScO_3_ undergoes critical behavior ≈275 K, below which a non‐zero SHG intensity is observed in this geometry (Figure 2c). In oblique incidence, the SHG intensity increases below ≈275 K reaching 10 × the room temperature intensity at 10 K. Following phase‐field predictions, the low‐temperature SHG polarimetry can be fitted to a multidomain monoclinic *m* model (see Supporting information), shown in the lower panel of Figure 2b for KNbO_3_ on GdScO_3_ at 10 K. Note that a multidomain orthorhombic *mm*2 symmetry can also be used to fit the polar plots at low‐temperature. Hence, to conclusively determine the symmetry of the low‐temperature phase, complementary temperature‐dependent x‐ray diffraction studies are necessary as discussed in Section 4.

The phase transition from tetragonal to a lower symmetry phase is further suppressed to ≈150 K for the KNbO_3_ on DyScO_3_ film (*ɛ* ≈ −1.6%), while for the highest strained KNbO_3_ on SrTiO_3_ (*ɛ* ≈ −2.7%), the low‐temperature phase is completely absent, as evidenced from the temperature‐dependence of normal and oblique incidence SHG amplitude as well as from the SHG polarimetry for both films (Figures S13 and S14, Supporting information). The observed phase transition temperatures are consistent with phase‐field predictions and reflect the influence of strain in favoring a tetragonal phase in KNbO_3_. Furthermore, it is noteworthy that the rhombohedral phase which appears in bulk KNbO_3_ below 225 K is completely suppressed in all these films, even for the lowest strained sample on GdScO_3_.

On the high‐temperature end, the strain‐stabilized tetragonal phase persists on heating up until 900 K for all three films, with no appreciable change in the measured SHG intensity. This suggests the robustness of the tetragonal phase. To test the highest operating temperature possible for the KNbO_3_ films, a KNbO_3_ film on GdScO_3_ was heated in air to temperatures higher than the film growth temperature (≈925 K). The film shows signs of decomposition evidenced by the declining SHG intensity when heated beyond ≈975 K, while still maintaining a tetragonal symmetry that persists when cooled back down to room temperature (Figure S15, Supporting Information).

By benchmarking the SHG polarimetry to a reference sample, nonlinear optical coefficients are derived using the #SHAARP.*ml* package,^[^ ^30^, ^31^ ^]^ and is discussed in further detail in Supporting Information. The measured *d*_33_ coefficient in strained tetragonal KNbO_3_ is shown in Figure 2d in reference to other similar ferroelectrics, reflecting a ≈200% enhancement of the *d*_33_ coefficient as compared to the bulk KNbO_3_ values while ≈50% higher than bulk PbTiO_3_ values.^[^ ^32^ ^]^ Since at low temperatures, the films have multiple domains, extracting the full SHG tensor at low temperatures is challenging. From multi‐domain modeling however, only a domain area‐fraction scaled *d*_11_ *d* coefficient can be estimated which is ≈44 pm V^−1^ at 10 K. Since the domain area‐fraction is strictly less than 1, this value should be considered only as a lower bound estimate of the low‐temperature *d*_11_ coefficient, i.e., *d*_11_ ≥ 44 pm V^−1^.

## 4 Structural Characterization by X‐Ray Diffraction

**Figure 3:**
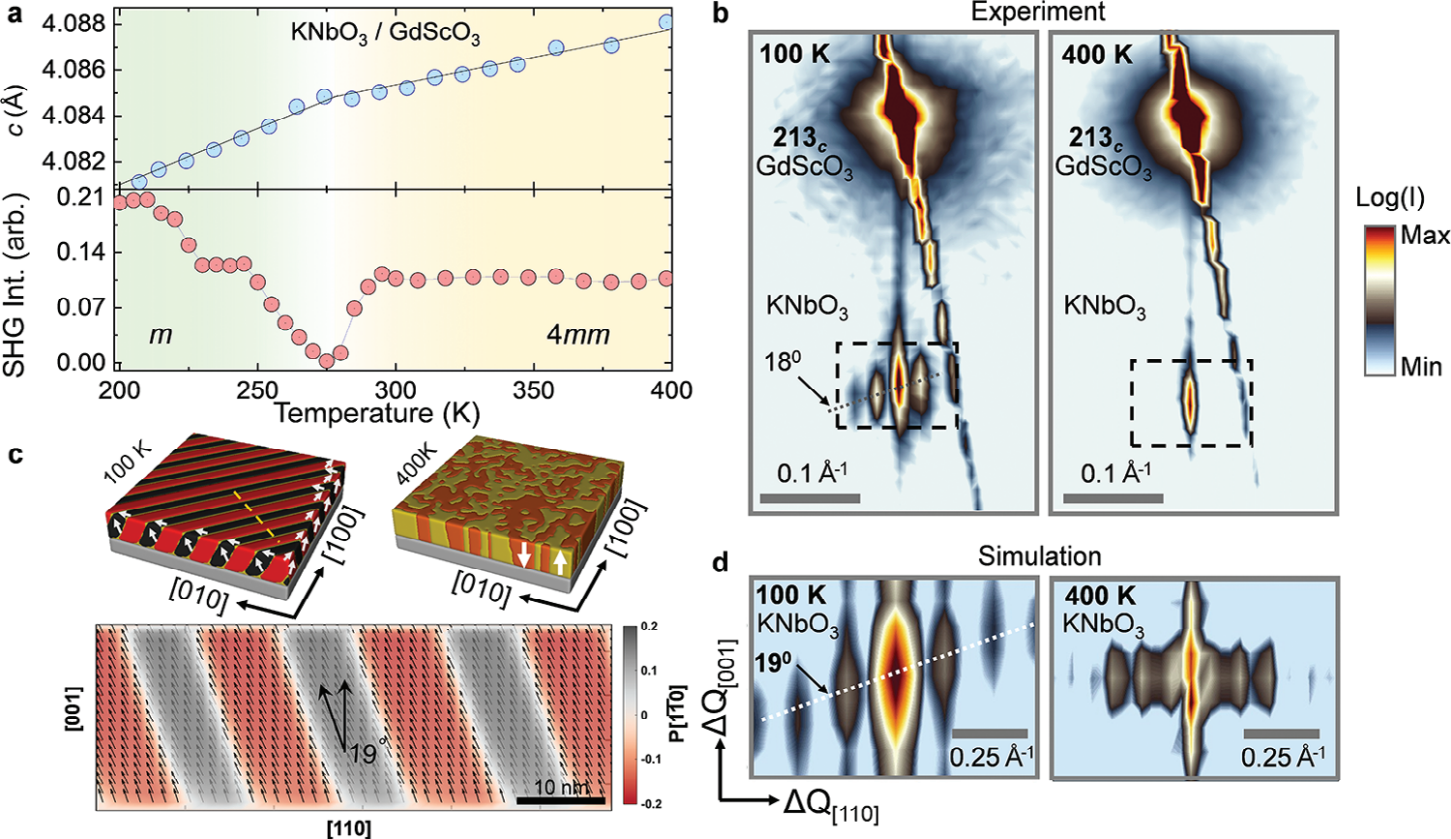
a) (Upper panel) Out‐of‐plane lattice parameter measured for the KNbO_3_ film grown on a GdScO_3_ substrate deduced from the position of the 002*_c_* specular peak between 200 K and 400 K, showing a change of the thermal expansion coefficient at 275 K. The phase transition temperature from the X‐ray agrees with the SHG measurements (lower panel, zoomed in from Figure 2c). b) Synchrotron‐based x‐ray reciprocal space maps (RSM) at 400 K and 100 K for the KNbO_3_ film grown on a GdScO_3_ substrate around the 213*_c_* (*c* stands for cubic) substrate Bragg reflection (tetragonal to monoclinic phase transition found from SHG at 275 K). The strong diagonal streak passing through the substrate peak is an artifact due to the spreading of the signal on the area detector for high intensity and is oriented along the Ewald sphere. Both at 400 K and 100 K, the films are coherent to the substrate but at 100 K, the film peak develops diffuse satellite peaks which are attributed to the formation of periodic monoclinic twin domains. The satellite peaks are tilted away from the in‐plane [110] direction toward the [001] direction by 17° ± 4° calculated by averaging over several peaks. The domains exhibit a 27 nm periodicity in the [110] direction extracted from the satellite peak separation. c) (left panel) Phase‐field simulated microstructure at 100 K showing monoclinic domains consistent with the domain pattern experimentally observed (left panel of (b)). A vertical cut of this microstructure is shown in the bottom panel along the [110] direction (bottom panel) revealing a tilt of the domain by 19° similar to experimental observations (left panel of (b)). Predicted domain periodicity is measured to be ≈22 nm through phase field simulations matching the experimental observation (≈27 nm). Phase field simulated microstructure at 400 K shows tetragonal domains with no in‐plane periodic structures (right panel) consistent with right panel of (b). d) Diffraction simulation of the phase field microstructure at 100 K and 400 K around the 213*_c_* KNbO_3_ peak (zoomed in to match q‐vectors corresponding to dotted boxs in panel (b)) showing a 19° tilt in the diffuse satellites at 100 K matching experimental observations whereas no tilt is seen corresponding to the simulation at 400 K.

To elucidate the structural phase transition to a lower symmetry phase upon cooling, the temperature‐dependent out‐of‐plane lattice parameter was measured for the KNbO_3_ thin film grown on GdScO_3_; this is the film with the lowest strain (−1.1%). As shown in **Figure** **3**a the KNbO_3_ film undergoes a change in its thermal expansion coefficient at 275 K, as extracted from the slope of lattice parameter variation versus temperature. This observation points to a film structure change that agrees with the SHG observations of the symmetry lowering at the same temperature of 275 K.

Additional 3D RSMs were obtained by synchrotron X‐ray diffraction that shed more light on the nature of these observations. Diffraction images were collected with a large area detector with an incident X‐ray photon energy of 50 keV, which allows sampling large reciprocal space volumes extending from 0 to 6.5 Å^−1^ in the out‐of‐plane direction and from −5 to 5 Å^−1^ in the in‐plane direction. In this configuration, the RSM measurements covered several peaks that are compared to each other to suggest a domain‐averaged tetragonal structure for the KNbO_3_ film (Figure S16, Supporting information). Unfortunately, the symmetry of the film structure may remain hidden in the domain‐averaged measurements. For this reason, the diffuse scattering patterns around selected peaks are more closely inspected that provide added information about the domain arrangements in the film. A 213*_c_* peak is chosen as a representative peak since this peak is sensitive to in‐plane polarization components along both [100] or [010] directions. For the case of KNbO_3_ on GdScO_3_ at 400 K, the 2D RSM of the film peak around the 213*_c_* GdScO_3_ peak (Figure 3b) shows film‐substrate commensuration where additional diffuse scattering patterns near the film peak could not be observed. In the case of low‐symmetry phases, we would have expected the formation of ferroelastic twins to generate diffuse scattering around the film position, whereas, in a tetragonal phase that is fully polarized out‐of‐plane as indicated by SHG measurements, the ferroelastic twin formation is not expected. Thus, X‐ray measurements are consistent with the SHG measurements and the phase‐field simulations in observing a tetragonal phase at >275 K. When cooled down below the phase transition temperature, the KNbO_3_ film peaks develop diffuse scattering patterns nearby, which correspond to the formation of twins with an in‐plane periodic arrangement. This reflects the symmetry lowering from the high‐temperature tetragonal structure to a low‐temperature monoclinic phase for the −1.1% biaxially strained thin films, supporting the SHG observations. The tilt of the diffuse satellite peaks can be directly correlated to the tilt of the monoclinic domains themselves relative to the substrate.^[^ ^33^, ^34^ ^]^ Averaging over several peaks (Figure S17, Supporting Information), this tilt is measured to be 17° ± 4° away from the [110] in‐plane direction toward the [001] out‐of‐plane direction, which agrees with the phase‐field microstructure modeling of monoclinic domains at 100 K, which shows a similar tilt of ≈19° away from the [110] direction (Figure 3c, left and bottom panels). Diffraction simulation of the phase‐field microstructure at 100 K (Figure 3d) also supports this expectation where diffuse satellites around the 213*_c_* KNbO_3_ peak show the same tilt of ≈19°, emphasizing the connection between the tilt of the satellite peaks and the tilt of the domains themselves. The same tilt is similarly observed for diffraction simulations around several other peaks as well (Figure S17, Supporting Information) which considerably strengthens our claim. In comparison, diffraction simulation of the phase‐field microstructure at 400 K (Figure 3d, right panel) shows diffuse scattering around the 213*_c_* KNbO_3_ peak without any tilt, consistent with the formations of only tetragonal domain variants with polarization along the [001] or [00$\bar{1}$] directions with 180° domain walls between them. The experimentally measured RSM at 400 K does not show such diffuse scattering patterns around the film peak. This is due to the absence of 180° tetragonal domains in these films. Only the polarization direction pointing upwards from the substrate appears to be stable as confirmed by piezoresponse force microscopy measurements (Section 5 and Figure S23, Supporting Information). Additionally, the experimentally observed diffuse satellite peaks at 100 K correspond to an in‐plane domain periodicity of ≈27 nm in agreement with the ≈22 nm periodicity predicted by phase field modeling.

## 5 Ferroelectricity

To test the ferroelectric behavior of KNbO_3_, thin films were grown on SrTiO_3_ and DyScO_3_ substrates with a 15 nm thick SrRuO_3_ bottom electrode layer. RHEED and RSMs were performed to confirm the crystallinity and commensurate strain nature of the films on the substrate (Figures S18 and S19, Supporting Information). Cross‐sectional TEM of KNbO_3_/SrRuO_3_/SrTiO_3_ films shows a uniform interface structure without any extended defects suggesting high‐quality epitaxial growth (Figure S11, Supporting Information). Temperature‐dependent SHG on KNbO_3_ films with SrRuO_3_ bottom electrode shows no effect of SrRuO_3_ on the phase transition temperatures of the KNbO_3_ films (Figure S20, Supporting Information).

**Figure 4:**
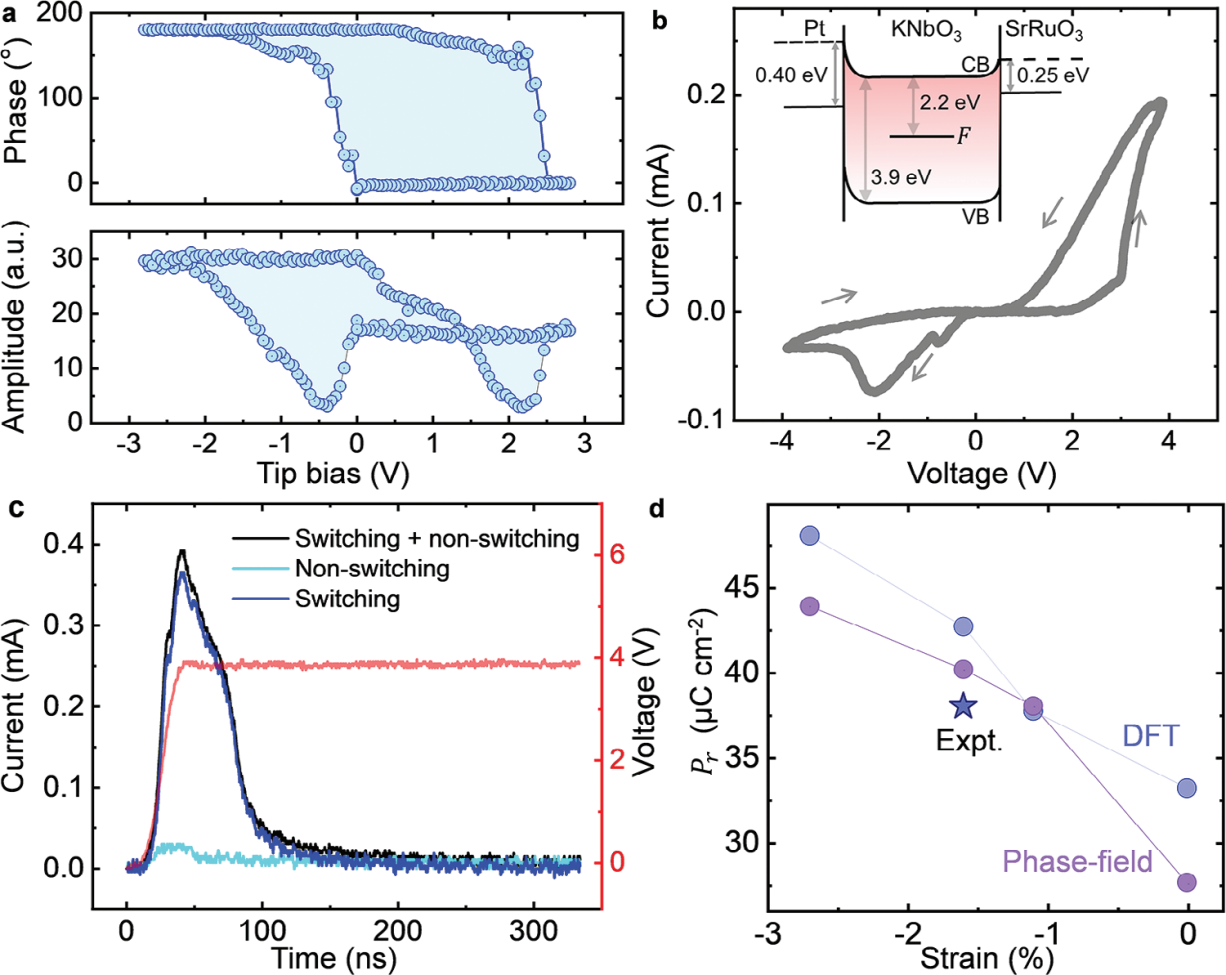
PFM switching spectroscopy and electrical measurements of the switching current on the 5 × 5 µm^2^ Pt/KNbO_3_/SrRuO_3_/DyScO_3_ capacitors. a) PFM switching spectroscopy measured at an arbitrary location on the capacitor shows standard ferroelectric *d*_33_‐*V* behavior. b) I–V measurement shows switching current peaks on the negative voltage side, while high leakage current smears out the switching peak on the positive side. The inset shows a schematic band diagram of KNbO_3_ depicting the measured Schottky barrier heights at both Pt‐KNbO_3_ and SrRuO_3_‐KNbO_3_ interfaces and the known *F*‐center (Oxygen‐vacancy which traps electrons) energy level in KNbO_3_.^[^ ^43^, ^44^ ^]^ c) PUND measurement of the switching peak at −4 V shows a switching time in the order of 80 ns and a remnant polarization of ≈38 µC cm^−2^. d) Comparison of polarization versus strain predicted from phase‐field simulations and DFT calculations along with experimentally observed value from PUND measurements. All values are for the tetragonal phase of KNbO_3_.

Piezoresponse force microscopy (PFM) and electrical measurements of the switching current were performed on the Pt/KNbO_3_/SrRuO_3_/DyScO_3_ capacitors with 21.8 nm thick KNbO_3_. **Figure** **4**a shows a representative PFM switching spectroscopy loop obtained at an arbitrary location on the capacitor at a quasi‐static frequency of 0.2 Hz, which exhibits standard ferroelectric *d*_33_‐*V* behavior with coercive voltages of −0.5 and 2.2 V. The films appear to be self‐poled in the as‐grown state with a remanent polarization, *P_r_*, pointing from the bottom to the top of the film. When poled down by applying a bias using the PFM tip, the strong upward built‐in field (≈0.8 MV cm^−1^) causes the downward polarization to relax back to the upward state in seconds (Figures S21–S23, Supporting Information). *I–V* measurements (Figure 4b) at a frequency of 100 kHz from the capacitors show clear switching current peaks on the negative voltage side, while a high leakage current appears on the positive voltage side obscuring the switching current signal. Additional testing shows that the high leakage on the positive voltage side increases with time. In addition, it can also be seen in Figure 4b that the current for the voltage decreasing from 4 to 0 V is higher than that for the voltage increasing from 0 to 4 V. This leakage, which cannot be subtracted using a standard PUND (positive‐up‐negative‐down) method,^[^ ^35^ ^]^ prevents measurements of the switching current on the positive bias side. In contrast, the switching current can be measured on the negative bias side using the PUND method (Figure 4c). The extracted switching current peak shows a switching time for the 5 × 5 µm^2^ capacitors on the order of 80 ns and a *P_r_* of ≈38 µC cm^−2^. Nonetheless, further investigation reveals that there is a dynamic leakage current during the polarization switching process that is not subtracted with the standard PUND method (Figure S22, Supporting Information), which suggests that the calculated polarization value is perhaps overestimated.^[^ ^36^, ^37^ ^]^ Polarization switching in KNbO_3_ films on DyScO_3_ and SrTiO_3_ substrates with SrRuO_3_ bottom electrode have also been confirmed with PFM measurements (Figure S21, Supporting Information), where the presence of the strong upward built‐in fields causes the polarization down state to be very unstable, and the as‐grown films are preferentially in the upward single domain states. Figure 4d shows a comparison of the measured *P_r_* with predicted *P_r_* values from phase field simulations and PBEsol calculations, indicating further room for the enhancement of the experimental *P_r_*.

Our current PFM measurements on the Pt/KNbO_3_/SrRuO_3_ capacitors show asymmetric leakage currents for opposite polarities. To understand the origin of the leakage current, temperature‐dependent IV measurements under DC bias were performed on Pt/KNbO_3_/SrRuO_3_/SrTiO_3_ capacitors. The IV measurements can be fitted to a Schottky emission model (Figures S24 and S25, Supporting Information), suggesting electrode‐film interface‐controlled Schottky emission to be one of the dominant contributors of leakage in KNbO_3_ films. The estimated Schottky barrier height is 0.4 ± 0.04 eV for the Pt/KNbO_3_ interface and 0.25 ± 0.03 eV for the SrRuO_3_/KNbO_3_ interface. The asymmetric Schottky barrier height is responsible for the asymmetric leakage current and is due to the different work functions of Pt (5.6 eV^[^ ^38^ ^]^) and SrRuO_3_ (5.2 eV^[^ ^39^ ^]^) electrodes and different interface chemistry at each interface (Figure 4b).

To further explore the dielectric response and additional charge transport mechanisms in the KNbO_3_ film, temperature, and frequency‐dependent modulus spectroscopy measurements were performed. The frequency‐dependent imaginary part of the electric modulus shows a dielectric relaxation peak with a corresponding Arrhenius activation energy of 0.45 ± 0.04 eV (Figure S27, Supporting Information). We hypothesize that this relaxation process is related to the polaron hopping mechanism which has previously been observed in KNbO_3_ single crystals.^[^ ^40^, ^41^ ^]^ Due to high K vapor pressure, K vacancies are common in KNbO_3_, which subsequently leads to oxygen vacancies. Since these oxygen vacancies are necessary for charge neutrality, they are thermodynamically stable and cannot be removed via an annealing process. Oxygen vacancies can also be formed due to oxygen leaving lattice positions serving as electron donor defects leading to carrier injection in KNbO_3_.^[^ ^42^ ^]^ Oxygen vacancies can contribute to both electronic and ionic conductivity in KNbO₃ films. Oxygen vacancies in KNbO_3_ can also serve as electron trapping sites (*F*‐center) which produces deep energy levels, ≈2.2 eV below the conduction band.^[^ ^43^, ^44^ ^]^ Finally, migration of K^+^ ions and oxygen vacancies (activation energies of 0.6 and 1.2 eV respectively)^[^ ^45^ ^]^ and the formation of conductive domain walls and pinholes across a thin film can also contribute to leakage in KNbO_3_ thin films.

Our present investigations in strain‐tuned KNbO_3_ films present a unique opportunity in KNbO_3_ where strain can not only be employed to enhance ferroelectric properties in KNbO_3_ but also these properties can be accessed stably across a wide temperature range of operation by pushing out phase transitions which would otherwise lead to divergence of ferroelectric properties. Though further work is necessary to understand the nature and role of intrinsic defects in KNbO_3_ thin films, SHG experiments show the robustness of KNbO_3_ films at high temperatures ranging up to ≈975 K. Notably, surface capping of KNbO_3_, the development of KNbO_3_‐based superlattice heterostructures, and slightly doping the film with electron acceptors would not only further enhance the stability of KNbO_3_ from the formation of intrinsic K and O vacancy point defects, but also would allow the growth of thicker coherently strained films avoiding mechanical sources of leakage like pinhole formations.

## 6 Conclusion

In summary, our experiments and simulations demonstrate that biaxially strained KNbO_3_ films result in the stabilization of a single tetragonal phase throughout the entire temperature range from 10 K to 975 K in contrast to four competing phases observed in bulk single crystals; this is challenging to achieve in other competing Pb‐free ferroelectrics such as BaTiO_3_ and Na_x_K_1‐x_NbO_3_. Concomitantly, a large enhancement in the tetragonality through strain is observed with its *c/a* ratio reaching values that exceed even those of bulk PbTiO_3_. A ≈200% increase in the tetragonal phase optical second harmonic generation coefficients over bulk single crystal values is observed. A dramatic *T_c_* enhancement well beyond its decomposition and melting temperatures are seen with a 3 × higher tunability, $\frac{dT_{c}}{d\varepsilon }$, as compared to all other strain‐tuned perovskite oxides. A broad design trend gleaned from this study is that for tetragonal perovskite ferroelectrics, a high *T_c_* is achieved by maximizing electrostriction and minimizing elastic compliance (Figure S3, Supporting information). Importantly, there are optimal values of electrostriction and compliance that maximize *P_r_*. For KNbO_3_, its superior electrostriction and low elastic stiffness coefficients compared to similar materials result in a large and highly strain‐tunable *T_c_* in addition to a competitive magnitude and tunability in *P_r_*. All these observations render strained KNbO_3_ thin films as a strong, thermally stable lead‐free ferroelectric with a large operating temperature regime suitable for a wide range of applications.

## 7 Experimental Section

### Thin Film Synthesis and Characterization

Epitaxial thin films of KNbO_3_ were grown using a Vecco Gen 10 MBE system. A molecular beam of NbO_2_ (gas) flux was generated from an effusion cell containing Nb_2_O_5_ (H.C. Stark 99.99%) contained in an iridium crucible. NbO_2_ is the most volatile species in the growth temperature range.^[^ ^46^ ^]^ Potassium was evaporated from an effusion cell containing an indium‐rich (≈4:1 In:K ratio) mixture of potassium and indium so it forms the air‐stable intermetallic In4K.^[^ ^47^ ^]^ The K‐In alloy was prepared in a glove box and contained in a titanium crucible. Once prepared, it can be exposed to air, facilitating its handling and loading. The vapor pressure of potassium was more than 10^10^ times higher than indium at the K‐In cell temperature of 300 ‐ 400 °C.^[^ ^48^ ^]^ GdScO_3_ (110)_o_, and DyScO_3_ (110)_o_ (Crystec GmbH) substrates were used as received and the SrTiO_3_ (001) substrates were terminated following the procedure developed by Koster.^[^ ^49^ ^]^

Films were grown by co‐deposition of potassium, niobium, and ozone at a substrate temperature of 700–650 °C as measured by an optical pyrometer operating at a wavelength of 1550 nm. The pyrometer measures the temperature of the platinum coating that had been evaporated on the backside of the substrate to facilitate radiative heat transfer from the SiC heating element of the MBE system to the substrate. The K:Nb flux ratio was kept at ≈10:1. A mixture of ozone and oxygen (10% O_3_ + 90% O_2_) was used as the oxidant. The films were grown at an oxidant background pressure of 1 × 10^−6^ Torr. Typical fluxes for the sources were (1 − 2) × 10^13^ atoms cm^−2^ s^−1^ for NbO_2_ and (1 − 4) × 10^14^ atoms cm^−2^ s^−1^ for potassium, determined by a quartz crystal microbalance (QCM), with an accuracy of ≈15%. In a typical growth experiment, the potassium flux was measured first, followed by NbO_2_ to ensure that the QCM was as close to room temperature as possible for the most accurate reading. For a more detailed description, the reader was referred to an identical procedure reported for KTaO_3._^[^ ^50^ ^]^ Co‐deposition with these fluxes results in a KNbO_3_ film growth rate of ≈0.3 Å s^−1^.

X‐ray diffraction (XRD), X‐ray reflectometry (XRR), and RSM measurements were carried out using a PANalytical Empyrean diffractometer with Cu *K*α_1_ radiation. The raw XRR spectra were analyzed using the PANalytical X´Pert Reflectivity software package and the layer thickness was derived from a fast Fourier transform (FFT) after manually defining the critical angle to account for refractive effects. In situ reflection high‐energy electron diffraction (RHEED) patterns were recorded using KSA‐400 software and a Staib electron source operated at 14 kV and a filament current of 1.5 A. The morphology of the film surface was characterized using an Asylum Cypher ES environmental AFM.

### Transmission Electron Microscopy

The cross‐sectional samples for scanning transmission electron microscopy (STEM) were prepared using a standard focused ion beam lift‐out process. The KNbO_3_ thin film on the SrTiO_3_ substrate was milled using the FEI Helios NanoLab 600 DualBeam, while the samples on GdScO_3_ and DyScO_3_ substrates were milled using the Helios G4 UX DualBeam system. All samples were initially thinned with 5 kV Ga ions and then polished at 2 kV to minimize surface damage. HAADF‐STEM images of KNbO_3_ samples on GdScO_3_ and DyScO_3_ were obtained using the Spectra 300 X‐CFEG microscope with a 60–200 mrad HAADF detector. For the KNbO_3_ film on SrTiO_3_, the Thermo Scientific Themis Z S/TEM with a 64–200 mrad HAADF detector was used. Both microscopes operated at 200 kV with a semi‐convergence angle of 30 mrad. To improve the signal‐to‐noise ratio, HAADF‐STEM images were captured as a series of 20 fast scan images (2048 × 2048 pixels, 200 ns per frame) and then averaged them. STEM energy‐dispersive X‐ray (EDX) spectroscopy data for films on GdScO_3_ and DyScO_3_ were collected using a steradian Dual‐X EDX detector. The resulting spectrum was then denoised using principal component analysis.

### Second Harmonic Generation (SHG) Measurement

SHG polarimetry and temperature‐dependent measurements were done with femtosecond pulses at *λ* = 800 nm fundamental light from a regeneratively amplified Spectra‐Physics Solstice Ace Ti:Sapphire laser system (1kHz, 100 fs). The schematic of the setup is shown in Figure 2a. Linearly polarized light incident on the sample at an incidence angle θ generate second harmonic light at *λ* = 400 nm. The *p*‐polarized and *s*‐polarized SHG intensities were spectrally filtered and measured by a photomultiplier tube through lock‐in amplifier (SR830) detection. Polar plots were generated by rotating the polarization angle (φ) of the incident fundamental light by a half‐wave plate. Temperature‐dependent measurements were done through a Janis 300 gas flow cryostat for low‐temperature and a home‐built heating stage for high‐temperature experiments.

### Synchrotron‐Based Reciprocal Space Mapping

Synchrotron x‐ray diffraction experiments were performed on the ID4B (QM2) beamline at the Cornell high energy synchrotron source (CHESS). The incident X‐ray energy was 50 keV (*λ* = 0.247 Å), which was selected using a double‐bounce diamond monochromator. An area detector array (Pilatus 6M) was used to collect the scattering intensities in a reflection geometry. The sample was rotated with a 1° tilt through 180° rotations, sliced into 0.1° frames. Geometric parameters of the Pilatus 6M detector such as detector distance, tilting, rotation, and direct beam position were extracted using standard CeO_2_ powder. The uncertainty in temperature was <10 K.

### X‐Ray Photoelectron Spectroscopy

The XPS experiment was performed using the Physical Electronics VersaProbe III instrument with a monochromatic Al *K*α x‐ray source (*h*ν = 1486.6 eV) and a concentric hemispherical analyzer. Charge neutralization was performed using low‐energy electrons (<5 eV) and argon ions. The binding energy axis was calibrated using sputter‐cleaned Cu (Cu 2p_3/2_ = 932.62 eV, Cu 3p_3/2_ = 75.1 eV) and Au foils (Au 4f_7/2_ = 83.96 eV). Measurements were made at a takeoff angle of 45° with respect to the sample surface plane. This resulted in a typical sampling depth of 3–6 nm. On homogeneous samples, major elements (>5 atom%) tend to have standard deviations of <3%, while minor elements can be significantly higher. The analysis size was 200 µm in diameter.

### PFM Spectroscopy Measurements

The PFM switching spectroscopy data were measured using an atomic force microscopy system (MFP3D, Asylum Research) with Pt‐coated tips (PPP‐EFM, Nanosensors) in the resonance enhanced mode (DART) with AC‐modulation voltage of 0.2 V at 340 kHz. DC voltage was applied to the top electrodes in the pulsed mode, where the pulse‐on period is for polarization switching and the pulse‐off period is for PFM signal detection.

### Pt Top Electrodes Deposition

The Pt top electrodes with a thickness of ≈15 nm were deposited ex situ by pulsed laser deposition (PLD) in vacuum at room temperature. 5 × 5 µm^2^ electrode patterns were prepared using a standard liftoff approach with a laser lithography system (Heidelberg DWL 66FS).

### PUND and I–V Measurements

A function generator (Keysight 33621A) was used for voltage pulse generation, and an oscilloscope (Tektronix TDS 3014B) was used for recording the switching current signal. Standard triangular waves at 100 kHz were used for the *I*–*V* measurements, and square pulses of 500 ns in width with one negative pulse followed by two positive and then two negative pulses were used for the PUND measurements (see Figure S17, Supporting Information). The pulse rise time is ≈20 ns. All voltages were applied to the top electrodes through the PFM tip.

### Leakage Current and Modulus Spectroscopy Measurements

To investigate the dominant conduction mechanisms responsible for the rise in leakage current in KNbO₃ films, leakage current measurements were performed on 21.8 nm thick KNbO_3_ films grown on DyScO_3_ with a 15 nm thick SrRuO_3_ bottom electrode, using a 4140 Pico‐Ampere Meter/DC Voltage Source (Hewlett Packard). 100 nm thick Pt electrodes which were lithographically patterned into 50 µm × 50 µm squares, were used as the top electrode.

To further explore additional charge transport mechanisms in the KNbO_3_ film, temperature‐dependent modulus spectroscopy measurements were performed using a Solartron 1260 Impedance analyzer with a l00 mV AC amplitude, over a frequency range from 1 MHz to 0.01 Hz.

### Phase‐Field Modeling

Using the phase field method, the evolution of the ferroelectric polarization (*P_i_*) was governed by the time‐dependent Ginzburg Landau equation:

(2) $$\gamma_{ij}\;\frac{\partial P_{j}\left(x_{i},\;t\right)}{\partial t}=\frac{\delta F}{\delta P_{i}\left(x_{i},t\right)}$$

where γ_*ij*_ is the kinetic coefficient tensor which is chosen to be isotropic and *F* is the total free energy of the system which is the integral of all energy density components:

(3) $$F=\underset{V}{\;\int \int \int \;}\left[f_{landau}\left(P_{i}\right)+f_{grad}\left(\nabla P_{i}\right)+f_{elas}\left(P_{i},\varepsilon_{ij}\right)+f_{elec}\left(P_{i},E_{i}\right)\right]\;dV$$

where *f_landau_*, *f_grad_*, *f_elas_* and *f_elec_* denote the Landau free‐energy density, the gradient energy density, the elastic energy density, and the electrostatic energy density respectively, and *V* is the volume of the system.

The Landau free energy density describes the intrinsic stability of the ferroelectric phases compared to the high symmetry phase (*m* $\bar{3}$ *m*) as a Taylor expansion of the polarization about the high symmetry phase:

(4) $$\begin{array}{ccc} f_{landau}\left(P_{i}\right) & = & \alpha_{1}\;\left(T\right)\left(P_{1}^{2}+P_{2}^{2}+P_{3}^{2}\right)+\alpha_{11}\left(P_{1}^{4}+P_{2}^{4}+P_{3}^{4}\right)\\ & & +\alpha_{12}\left(P_{1}^{2}P_{2}^{2}+P_{1}^{2}P_{3}^{2}+P_{2}^{2}P_{3}^{2}\right)+\alpha_{111}\left(P_{1}^{6}+P_{2}^{6}+P_{3}^{6}\right)\\ & & +\alpha_{112}\left(P_{1}^{4}\left(P_{2}^{2}+P_{3}^{2}\right)+P_{2}^{4}\left(P_{1}^{2}+P_{3}^{2}\right)+P_{3}^{4}\left(P_{1}^{2}+P_{2}^{2}\right)\right)\\ & & +\alpha_{123}P_{1}^{2}P_{2}^{2}P_{3}^{2}+\alpha_{1111}\left(P_{1}^{8}+P_{2}^{8}+P_{3}^{8}\right)+\alpha_{1112}\left(P_{1}^{6}\left(P_{2}^{2}+P_{3}^{2}\right)\right.\\ & & \left.+P_{2}^{6}\left(P_{1}^{2}+P_{3}^{2}\right)+P_{3}^{6}\left(P_{1}^{2}+P_{2}^{2}\right)\right)\\ & & +\alpha_{1122}\left(P_{1}^{4}P_{2}^{4}+P_{2}^{4}P_{3}^{4}+P_{3}^{4}P_{1}^{4}\right)\\ & & +\alpha_{1123}\left(P_{1}^{4}P_{2}^{2}P_{3}^{2}+P_{1}^{2}P_{2}^{4}P_{3}^{2}+P_{1}^{2}P_{2}^{2}P_{3}^{4}\right) \end{array}$$

The Landau expansion coefficients were adjusted from the work of Liang^[^ ^11^ ^]^ to fit with experimentally measured polarization and dielectric properties.^[^ ^51^, ^52^, ^53^ ^]^ The revised set of coefficients is listed under Table S1 in Section S1 (Supporting Information).

The gradient energy density is represented by:

(5) $$f_{grad}=\frac{1}{2}\;G_{ijkl}\frac{\partial P_{i}}{\partial x_{j}}\frac{\partial P_{k}}{\partial x_{l}}$$

where *G_ijkl_* is the gradient energy tensor where the non‐zero coefficients are chosen to be *G*_11_ =  0.6, *G*_22_ =   − 0.6 and *G*_44_ =  0.6 and the units are normalized by $\alpha_{1}l_{o}^{2}$ where α_1_ is the first Landau expansion coefficient and *l_o_* is chosen as 1 nm per grid.

The elastic energy density is calculated as:

(6) $$f_{elas}\;\left(P_{i},\;\varepsilon_{ij}\right)=\frac{1}{2}\;c_{ijkl}\left(\varepsilon_{ij}-\varepsilon_{ij}^{o}\right)\left(\varepsilon_{kl}-\varepsilon_{kl}^{o}\right)$$

where *c_ijkl_* represents the stiffness tensor, ɛ_*ij*_ is the total strain using the parent cubic phase as a reference, and $\varepsilon_{ij}^{o}$ is the eigenstrain which is dictated by the ferroelectric polarization and the electrostrictive tensor *Q_ijkl_* as:

(7) $$\varepsilon_{ij}^{o}=Q_{ijkl}\;P_{k}P_{l}$$

The elastic constant tensor components and electrostrictive coefficients used in this work are listed in Table S2 in Section S1 (Supporting Information).

The electrostatic energy density can be expressed as:

(8) $$f_{elec}\;\left(P_{i},\;E_{i}\right)=-E_{i}P_{i}-\frac{1}{2}\varepsilon_{o}\kappa_{ij}E_{i}E_{j}$$

where *E_i_* is the component of the electric field, ɛ_*o*_ is the dielectric permittivity of vacuum and κ_*ij*_ is the background dielectric susceptibility which accounts for the contributions to the total dielectric susceptibility beyond the ferroelectric soft mode following the work of Tagentsev.^[^ ^54^ ^]^

The evaluation of the elastic and electrostatic energy contributions in the phase‐field method could be found in the existing literature.^[^ ^55^, ^56^, ^57^ ^]^ At each time step, the electrical and mechanical equilibrium equations were solved under short‐circuit and thin‐film boundary conditions respectively.

For the simulations, a spatially discretized system of 128Δ*x* ×  128Δ*y* ×  36Δ*z* grids was employed with a grid spacing Δ*x*  =  Δ*y*  =  Δ*z*  = 1 nm. The film thickness was set to 20Δ*x*, and the substrate was 10Δ*x*, with a 4Δ*x* layer of air above the film. The in‐plane strains ɛ_11_ and ɛ_22_ were set equal to the misfit strains imposed by the lattice mismatch with the substrate. An initial random polarization distribution with a small magnitude of fluctuations of ∆*P *= 0.1 Cm^−2^ was started and let the system evolve to equilibrium.

The details on the construction of the phase diagram are provided in Note S1 (Supporting Information).

### Diffraction Simulation

The diffraction intensity (*I*) was calculated at the reciprocal space position ***q*** from:

(9) $$I\;\left(q\right)={\left|F\left(q\right)\right|}^{2}$$

where *F* is the structure factor obtained by:

(10) $$F\;\left(q\right)=\sum_{m,n}f_{n}e^{-q\cdot r_{m,n}}$$

where *m* is the unit cell index, *n* is the index of the atoms within the unit cell, and *f_n_* is the atomic form factor of the *n*‐th atom which is comprised of a real and imaginary part:

(11) $$f_{n}=f_{n}^{1}+if_{n}^{2}$$

The atomic form factors for the constituent atoms were obtained from the tabulated atomic data,^[^ ^58^ ^]^ assuming a photon energy of 10 KeV. The position of each atom (***r***_***m***, ***n***_) was calculated assuming a linear dependence of the atomic positions upon the polar order, i.e.,:

(12) $$r_{m,n}=R_{m,n\;}+s_{n}P_{m}$$

where ***R***_***m***, ***n ***_is the reference position in the absence of a polarization and ***s***_***n***_ are atomic site‐specific displacement coefficients relating the polarization to the atomic displacement. Atomic form factors and site‐specific displacement coefficients are given in Table S4 (Supporting Information).

### Density Functional Theory

The different phases were structurally optimized in the framework of density‐functional theory (DFT) with the open‐source software ABINIT.^[^ ^59^, ^60^, ^61^ ^]^ A plane‐wave basis set with a kinetic energy cutoff of 49 Ha was used to expand the wavefunctions. Optimized norm‐conserving Vanderbilt pseudopotentials from the PseudoDojo^[^ ^62^, ^63^ ^]^ (v0.4.1) were adopted and the exchange‐correlation energy was modeled using the Perdew–Burke–Ernzerhof generalized‐gradient approximation modified for solids (GGA‐PBEsol).^[^ ^64^, ^65^ ^]^ The Brillouin zone was sampled with a Monkhorst−Pack^[^ ^66^, ^67^ ^]^ 8 × 8 × 8 k‐point mesh and the self‐consistent field cycles were converged until the residual on the potential reached 10^−12^. The structures were relaxed until a maximum force of 2.5 meV Å^−1^ on each atom was reached. The local‐density approximation^[^ ^68^ ^]^ and the GGA‐PBE exchange correlation were also tested, but the PBEsol functional resulted in the best *c*/*a* ratio for the tetragonal and orthorhombic phases. For each phase, the in‐plane lattice parameters were fixed while the out‐of‐plane lattice parameter and the internal atomic positions were allowed to relax. For the monoclinic phase, since it presents too many degrees of freedom to adopt the same methodology, the in‐plane lattice parameters were set to the average values obtained by the phase‐field simulations at 25K for the two strains corresponding to the GdScO_3_ and DyScO_3_ substrates.

The polarization was computed with the Vienna ab initio simulation package (VASP v6.3.0).^[^ ^69^, ^70^, ^71^, ^72^ ^]^ The exchange‐correlation energy was modeled with the GGA‐PBE and the projector augmented wave method (PAW) method was used.^[^ ^73^ ^]^ The wavefunctions were expanded on a plane‐wave basis set with a kinetic energy cutoff of 680 eV. A 6 × 6 × 6 Γ‐centered k‐point mesh was adopted to sample the Brillouin zone, and the total energy was converged to 1 µeV in the electronic self‐consistent loops. The polarization calculations were automated with the atomate2 python package.^[^ ^74^ ^]^

## Conflict of Interest

The authors declare no conflict of interest.

## Supporting information



Supporting Information

## Data Availability

The data that support the findings of this study are available within the article. Additional data related to the film growth and structural characterization by XRD and STEM are available at https://doi.org/10.34863/fs5e-s772. Any additional data connected to the study are available from the corresponding author upon reasonable request.
