# Supplementary material for: Colossal Strain Tuning of Ferroelectric Transitions in KNbO3 Thin Films
Source: Adv Mater. 2024 Nov 12;36(52):2408664. doi: 10.1002/adma.202408664 (PMC11681320; doi:10.1002/adma.202408664)
Supplement: Supplementary file 1 — Supporting Information [file ADMA-36-2408664-s001.docx]

**Supporting Information**

**Colossal Strain Tuning of Ferroelectric Transitions in KNbO_3_ Thin Films**

*Sankalpa Hazra*, Tobias Schwaigert*, Aiden Ross, Haidong Lu, Utkarsh Saha, Victor Trinquet, Betul Akkopru-Akgun, Benjamin Z. Gregory,* *Anudeep Mangu, Suchismita Sarker, Tatiana Kuznetsova, Saugata Sarker, Xin Li, Matthew R. Barone, Xiaoshan Xu, John W. Freeland, Roman Engel-Herbert, Aaron M. Lindenberg,* *Andrej Singer, Susan Trolier-McKinstry, David A. Muller, Gian-Marco Rignanese, Salva Salmani-Rezaie, Vladmir A. Stoica, Alexei Gruverman, Long-Qing Chen, Darrell G. Schlom, and Venkatraman Gopalan*

**Note 1: Phase-field simulations**

**Table S1**. Set of Landau expansion coefficients

| Coefficient | Value | Unit |
| --- | --- | --- |
| $\alpha_{1}$ | $1.906\times{10}^{7}\left( \coth\left( \frac{54}{T(K)} \right)-\coth\left( \frac{54}{650} \right) \right)$ | [C^-2^ m^2^ N] |
| $\alpha_{11}$ | -5.86$\times$10^8^ | [C^-4^ m^6^ N] |
| $\alpha_{12}$ | 9.66$\times$10^8^ | [C^-4^ m^6^ N] |
| $\alpha_{111}$ | 2.71$\times$10^9^ | [C^-6^ m^10^ N] |
| $\alpha_{112}$ | -2.2$\times$10^9^ | [C^-6^ m^10^ N] |
| $\alpha_{123}$ | 4.4$\times$10^9^ | [C^-6^ m^10^ N] |
| $\alpha_{1111}$ | 1.74$\times$10^10^ | [C^-8^ m^14^ N] |
| $\alpha_{1112}$ | 5.99$\times$10^9^ | [C^-8^ m^14^ N] |
| $\alpha_{1122}$ | 2.5$\times$10^10^ | [C^-8^ m^14^ N] |
| $\alpha_{1123}$ | -1.63$\times$10^10^ | [C^-8^ m^14^ N] |

Modified from Liang et. al^[1]^ based upon experimental data from ^[2–4]^.

**Table S2.** Elastic stiffness coefficients and electrostrictive coefficients

| $c_{11}$ | 2.3$\times$10^11^ | [Pa] |
| --- | --- | --- |
| $c_{22}$ | 0.9$\times$10^11^ | [Pa] |
| $c_{44}$ | 0.76$\times$10^10^ | [Pa] |
| $Q_{11}$ | 0.11 | [C^-2^ m^4^] |
| $Q_{13}$ | -0.053 | [C^-2^ m^4^] |
| $Q_{44}$ | 0.052 | [C^-2^ m^4^] |

The phase diagram is established based on the polydomain structures obtained at equilibrium from phase-field simulations. The temperature is varied from 10 K to 1300 K at intervals of 25 K and the strain is varied from -4.5% to 0% at intervals of 0.1%. The phase classification in the phase diagram is performed based on the domain structure that exists at the end of a simulation.

The paraelectric region in the phase diagram marks the phase where (P_1_, P_2_, P_3_) $=$ 0. For the single-phase tetragonal, orthorhombic and rhombohedral regions, all the polarization vectors falling within the interior of a conical surface with the boundaries of the surface tilted 10**°** to the family of nominal crystallographic directions [001], [101] and [111] are classified in the single-phase regions, with the phase fraction in the classified phase greater than 0.95. For polarization vectors deviating more than 10**°** from the above nominal crystallographic directions, they are classified in the monoclinic *M_A_* and *M_C_* phases depending on the minimum angle created with the family of monoclinic planes (110) or (100) with phase fraction greater than 0.95.

The visualization of the domain structures from phase-field simulations also confirms the classified single phases with the remaining phase fraction of the polarization vectors in these single-phase regions formed in the domain walls separating two domain variants of the same phase, e.g., orthorhombic [101] and orthorhombic [10-1]. The remaining data points in the phase diagram not satisfying the above phase classification criteria are classified in either a two-phase mixture or a three-phase mixture depending on the phase fraction and the visualization of the domain structure.

**Table S3**: Comparison of $C$, $Q$, $S$ and $\frac{dT_{c}}{d\varepsilon}$coefficients for KNbO_3_ and related materials:

| Material | $C$ [K] | $Q_{11}$  [m^4^C^-2^] | $Q_{13}$ [m^4^C^-2^] | $S_{11}$  [m^2^N^-1^] | $S_{13}$  [m^2^N^-1^] | $\frac{dT_{c}}{d\varepsilon}$ *=*${4\epsilon}_{0}C\frac{\left( Q_{13} \right)}{\left( S_{11}+S_{13} \right)}$ |
| --- | --- | --- | --- | --- | --- | --- |
| SrTiO_3_^[5]^ | 0.505$\times$10^5^ | 0.0457 | −0.0135 | 3.8$\times$10^-12^ | -9.1$\times$10^-13^ | 83 K |
| PbTiO_3_^[6]^ | 1.5$\times$10^5^ | 0.089 | -0.026 | 8.0$\times$10^-12^ | -2.5$\times$10^-12^ | 251 K |
| BaTiO_3_^[7]^ | 1.37$\times$10^5^ | 0.10 | -0.034 | 9.0$\times$10^-12^ | -3.2$\times$10^-12^ | 284 K |
| KTaO_3_^[8,9]^ | 0.698$\times$10^5^ | 0.0872 | -0.023 | 2.5$\times$10^-12^ | -0.4$\times$10^-12^ | 280 K |
| KNbO_3_ | 1.6$\times$10^5^ | 0.11 | -0.053 | 5.6$\times$10^-12^ | -1.6$\times$10^-12^ | **750 K** |

**Table S4: Parameters used for diffraction simulations**

| Atom | $f^{1}$ | $f^{2}$ | $s$ |
| --- | --- | --- | --- |
| K | 19.3678 | 0.746466 | 1.01E-11 (m^3/C) |
| Nb | 40.5697 | 1.67439 | -0.56E-11 (m^3/C) |
| O | 8.035 | 0.021 | -3.15E-11(m^3/C) |


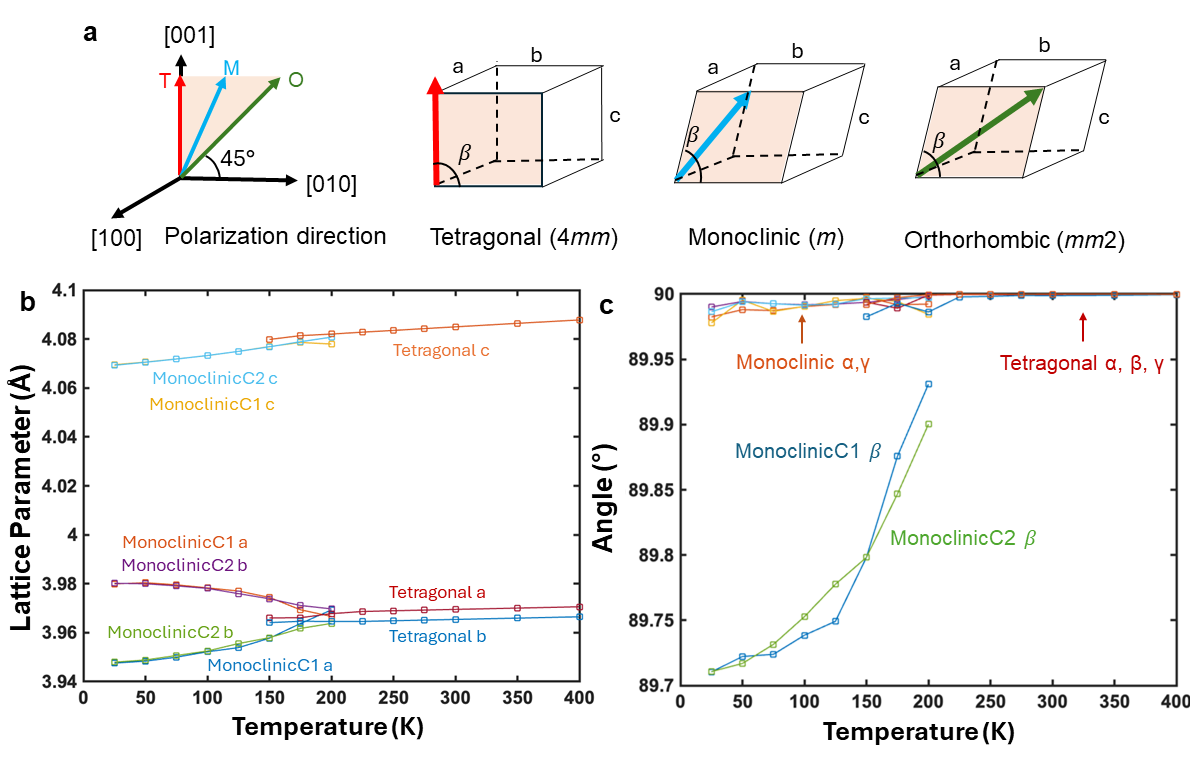


**Figure S1:** Schematic of polarization direction and unit cell corresponding to Tetragonal (4*mm*), Monoclinic (*m*) and Orthorhombic (*mm*2) phases observed in phase field simulations under compressive strains. Lattice parameters of KNbO_3_ on GdScO_3_ as a function of temperature across the tetragonal-to-monoclinic phase transition is shown in **(b)** and **(c)**. Monoclinic C1 and C2 refer to the two variants of the monoclinic phases stabilized from symmetry lowering from the Tetragonal phase. These two monoclinic unit cells represent shearing along [100] and [010] respectively. Lattice parameters and angles are all derived from phase field simulations.

The phase transitions seen from the phase field simulations are primarily driven by a reduction in the total free energy. In the case of ferroelectric domain structures under a substrate induced epitaxial strain, the main driving forces involve a competition between the intrinsic stability of the ferroelectric phase (Landau Energy) which favors the polarization along certain crystallographic directions and the elastic energy which aligns the polarization driven by the misfit strain due to the lattice mismatch between the film and the substrate.

From the Landau energy, under stress-free conditions, we find that the cubic phase is stabilized above T_c_ = 435 $℃$, the tetragonal phase is stabilized from 225-435 $℃$, the orthorhombic phase is stabilized from -50-225 $℃$, and the rhombohedral phase is stable below -50 $℃$ ^[1]^. However, under thin-film boundary conditions^[10]^, these bulk phase boundaries will be shifted and the bulk phases themselves may become distorted to better compensate for the elastic boundary conditions.

The driving mechanism for the stabilization of the tetragonal phase with out-of-plane polarization under a compressive strain is primarily elastic in origin. Under stress free conditions, this tetragonal phase possesses an intrinsic compressive strain along its a-axis and tensile strain along its c-axis (using the high symmetry cubic phase as the strain zero reference state). In the compressive strain region, since the tetragonal phase best matches the strain conditions imposed by the substrate, it has a minimal elastic energy in comparison to the other possible phases. While the tetragonal phase with out-of-plane polarization is favored with increasing compressive strain, the lower symmetry phases become more stable at lower compressive strains < - 0.5% regime.

Under these strain conditions, distorted versions of lower symmetry bulk orthorhombic and rhombohedral phases are observed which are described by monoclinic phases with space group P*m* and C*m* respectively in our phase diagram.

The P*m* monoclinic phase occurs at strain values from ~ -1% to ~ -0.75% at room temperature with the phase boundaries shifting towards higher compressive strains at lower temperatures. Conceptually, this monoclinic phase (**Figure S1a**) can be viewed as a distorted orthorhombic phase, where the compressive strain causes the polarization to rotate away from the 45° angle seen in the bulk orthorhombic phase towards the c-axis. This monoclinic phase has multiple domain variants resulting in a twinned domain microstructure seen in **Figure 3c, Main text**. **Figure S1b and c** show the change of unit cell lattice parameters as function of temperature predicted for KNbO_3_ on GdScO_3_ (*ε* ~ -1.1%) showing the structural phase transition from a tetragonal (P4*mm*) to monoclinic (P*m*) structure.

The C*m* monoclinic phase occurs at a strain around ~ -0.25% at room temperature with the phase boundaries shifting towards higher compressive strains at lower temperatures. This monoclinic phase can be viewed as a distorted version of the bulk rhombohedral phase where the compressive strain causes the polarization to rotate towards the c-axis away from the [111] nominal direction.

Furthermore, these thin-film boundary conditions allow for the coexistence of multiple ferroelectric phases which may be understood from the theory of strain-phase decomposition^[11]^. For a system with strain inhomogeneity, i.e., different strains along the in-plane a and b axes, at a fixed temperature, the maximum number of possible coexisting phases is three according to the Gibbs phase rule. Considering the three possible phases as $\alpha$, $\beta$ and $\gamma$ with homogeneous strains $\varepsilon_{1}^{i}$ and $\varepsilon_{2}^{i}$ (*i* = $\alpha$, $\beta$, $\gamma$) at equilibrium, the triangular region of the common tangent plane of the free energy bounded by the three tangent points corresponds to the three-phase coexisting region in the ε_1_-ε_2_ diagram. For each two of the single-phase free energy surfaces, a set of common tangent planes, and the associated tangent points and lines can be identified. These lines link the local strains at equilibrium and constitute the two-phase regions in the ε_1_-ε_2_ phase diagram. If we only consider the biaxial strain, i.e., ε_1_ = ε_2_, we can construct a diagonal ε_1_ = ε_2_ line in the ε_1_-ε_2_ phase diagram which gives us the possible two-phase and three-phase coexistence regions at each temperature.

The temperature-strain phase diagram for biaxial compressive strains where the tetragonal phase with out-of-plane polarization is mainly favored over other phases as mentioned earlier due to the dominance of the elastic energy over other energy contributions. However, in the tensile strain region, above room temperature, in-plane orthorhombic or in-plane tetragonal phases are favored^[12]^ as these phases possess a spontaneous in-plane tensile strain and an out-of-plane compressive strain along the c-axis leading to the best match with the strain conditions imposed by the substrate and consequently, lower elastic energy compared to other phases.

**Note 2: Density functional theory simulation.**

**
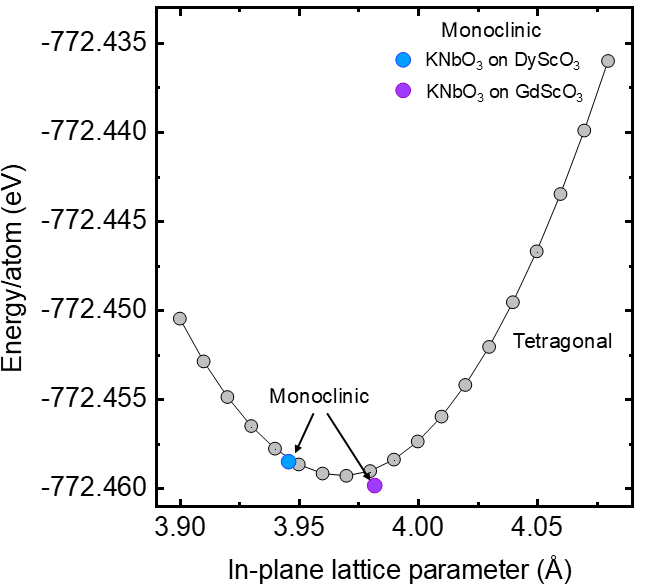
**

**Figure S2:** Atomic level density functional theory calculations (DFT) of total energy/atom showing the competitive stability of the tetragonal phase (grey curve) and monoclinic phase for KNbO_3_ on GdScO_3_ and KNbO_3_ on DyScO_3_ (blue and purple points).

For the tetragonal unit cell, the in-plane lattice parameter was fixed while the out-of-plane lattice parameter and internal atomic positions were allowed to relax. Hence, a continuous curve (grey) for the tetragonal structure could be extracted as a function in-plane lattice parameter. However, for monoclinic symmetry, the large number of degrees of freedom restricts the same methodology. For calculations for the monoclinic unit cell, the in-plane lattice parameters of KNbO_3_ on GdScO_3_ and DyScO_3_ substrates were adapted from phase field simulations at 25K for both samples. Hence, total energy/atom of the monoclinic unit cell could be calculated only at two distinct points.

.

From figure S2, although the monoclinic phase seems to be energetically favored over the tetragonal one, the difference in energy is less than 1 meV/atom, which is within the typical error margin of DFT. Hence, through DFT calculations the tetragonal and monoclinic phases are predicted to be comparable to one another.

It is important to note that, for calculations of total energy/atom our DFT does not account for multiple domain variants in the lower symmetry monoclinic phase, which has both been experimentally observed and predicted through phase-field simulations.

**Note 3: Dependence of** $\frac{\boldsymbol{d}\boldsymbol{T}_{\boldsymbol{c}}}{\boldsymbol{d\varepsilon}}$ **and** $\frac{\boldsymbol{d}\boldsymbol{P}_{\boldsymbol{r}}}{\boldsymbol{d\varepsilon}}$ **on electrostriction (**$\boldsymbol{Q}_{\boldsymbol{13}}$**) and elastic compliance (**$\boldsymbol{S}_{\boldsymbol{11}}\boldsymbol{+}\boldsymbol{S}_{\boldsymbol{13}}$**) coefficients**.

**
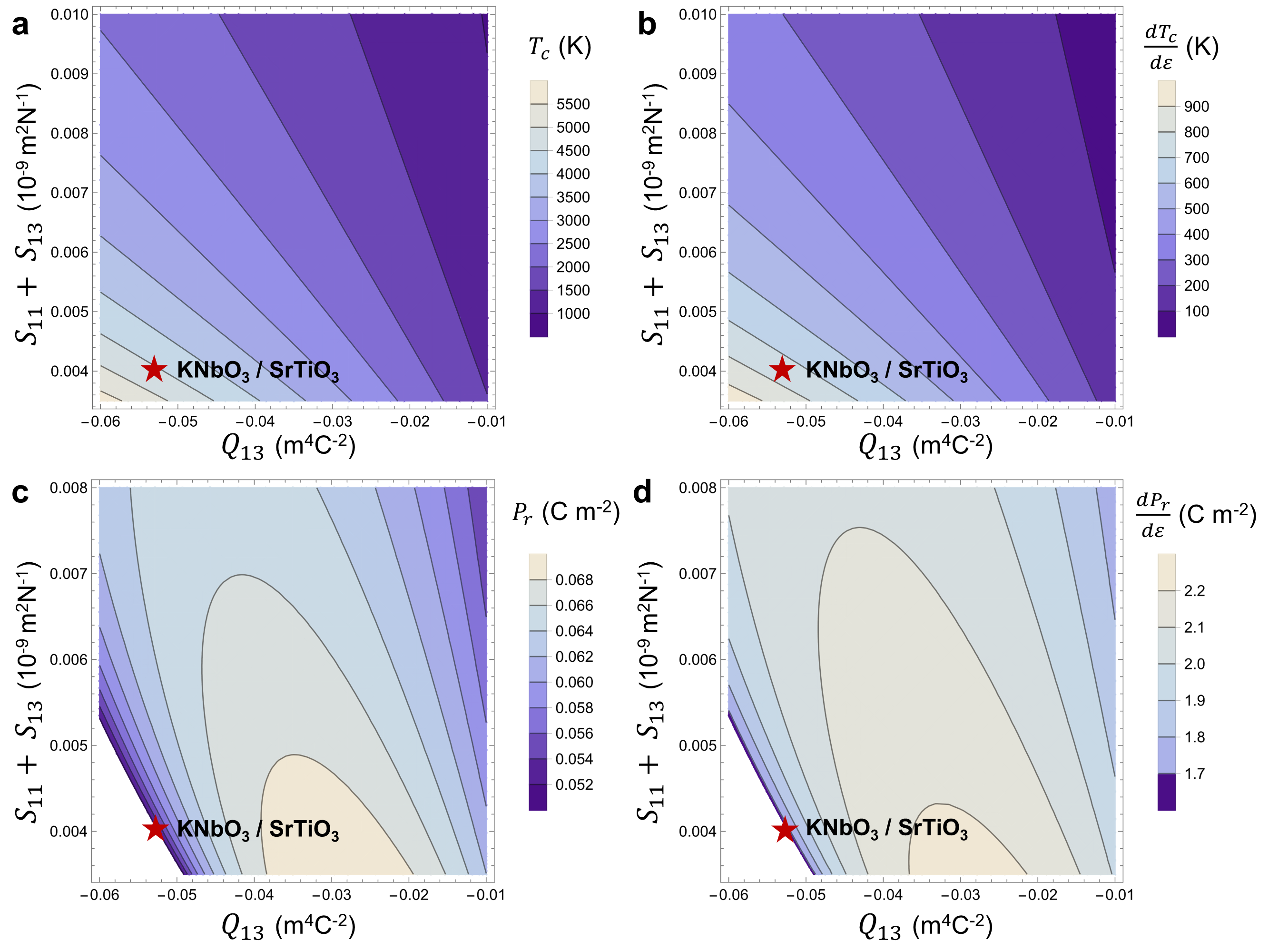
**

**Figure S3:** Contour plot showing the dependence of **(a)** $T_{c}$, **(b)** $\frac{dT_{c}}{d\varepsilon}$, **(c)** $P_{r}$, **(d)** $\frac{dP_{r}}{d\varepsilon}$ on electrostriction ($Q_{13}$) and elastic compliance ($S_{11}+S_{13}$) coefficients for compressively strained tetragonal phase calculated from analytical thermodynamic expressions, where any other relevant tensor coefficients are assumed to be the same as for KNbO_3_ (**Table S1 Supporting Information**). The white regions in panel c and d represent points where no real solution of the polarization exists. The red star on each panel denotes KNbO_3_ on SrTiO_3_ film, showing that the low ($S_{11}+S_{13}$) and high $Q_{13}$ values of KNbO_3_ render a high $T_{c}$ and $\frac{dT_{c}}{d\varepsilon}$, value, however $P_{r}$ and $\frac{dP_{r}}{d\varepsilon}$ are lower than maximum achievable.

**Note 4: Growth of strained KNbO_3_ thin films by suboxide molecular beam epitaxy**

Epitaxial KNbO_3_ thin films were grown on (001) SrTiO_3_, (110)_o_ DyScO_3_ and (110)_o_ GdScO_3_ substrates by suboxide molecular beam epitaxy with in-situ high pressure reflection high energy electron diffraction (RHEED). RHEED was used to monitor the evolution of surface structure reconstruction during growth. **Figure S4a-c** show RHEED patterns along the high symmetry directions where diffractions streaks and Kikuchi lines are visible for GdScO_3_ (110)_o_, DyScO_3_ (110)_o_ and SrTiO_3_ (001) substrates during the deposition of the first monolayer of KNbO_3_. **Figure S4d-f** show the KNbO_3_ RHEED streaks immediately after the deposition was stopped, where the shutter of the NbO_2_ and potassium source have been closed, but still at growth temperature (650 °C) and immersed in ozone. Atomic force microscopy (AFM) images are shown in **Figure S5** at different magnifications. Atomic steps are visible from the 0.1° off-cut GdScO_3_ and SrTiO_3_ are visible. The root-mean-square (rms) roughness for KNbO_3_ (001) on GdScO_3_ (110)_o_ (**Figure S5d)** is 0.55 nm, KNbO_3_ (001) on DyScO_3_ (110)_o_ (**Figure S5e**) is 0.61 nm and KNbO_3_ (001) on SrTiO_3_ (001) is 0.74 nm (**Figure S5f**).

**Figure S6a** shows the X-ray reciprocal space mapping (RSM) around the GdScO_3_ (332) DyScO_3_ (332) and SrTiO_3_ (103) reflections confirm that the KNbO_3_ films are commensurately strained to the respective substrates. **Figure S6b** compares the expected and measured out-of-plane lattice constants for KNbO_3_ films grown on different substrates. **Figure S6c** shows the $\theta-2\theta$ x-ray diffraction (XRD) scans for all three films. The rocking curves full width at half maximum (FWHM) of the KNbO_3_ films and the GdScO_3_ (110)_o_, DyScO_3_ (110)_o_ and SrTiO_3_ (001) substrates are shown in **Fig. S7**. FWHM are comparable to that of the respective substrates and increase with lattice mismatch between the substrate and the film. For the “cube-on-pseudocube” substrates (GdScO_3_ and DyScO_3_), rocking curves are collected along the two orthogonal in-plane directions of the substrate.


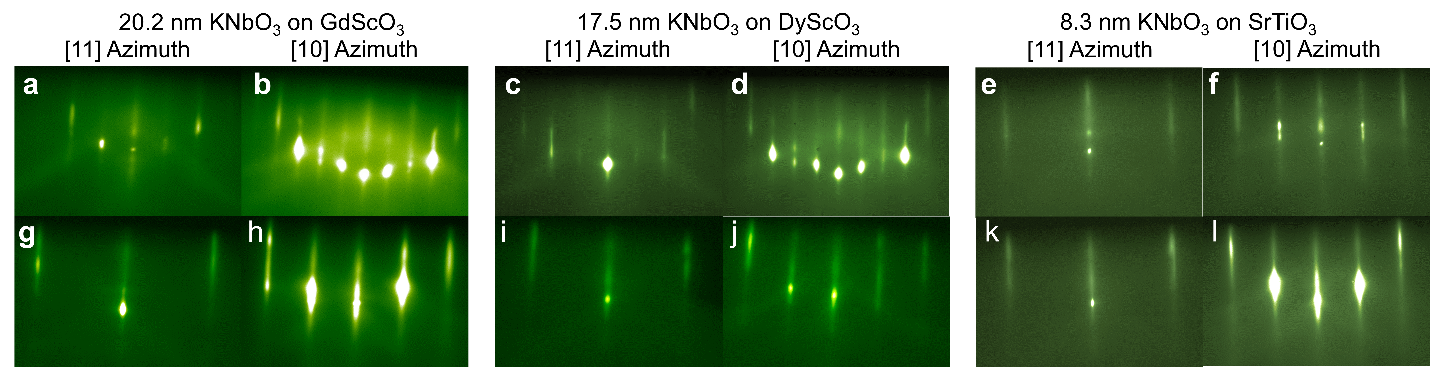


**Figure S4**: RHEED patterns of KNbO_3_ films grown on **a.** GdScO_3_, **b.** DyScO_3_ and **c** SrTiO_3_ substrates. The top row depicts the deposition of the first unit-cell and the bottom row represents the patterns immediately after deposition was stopped.


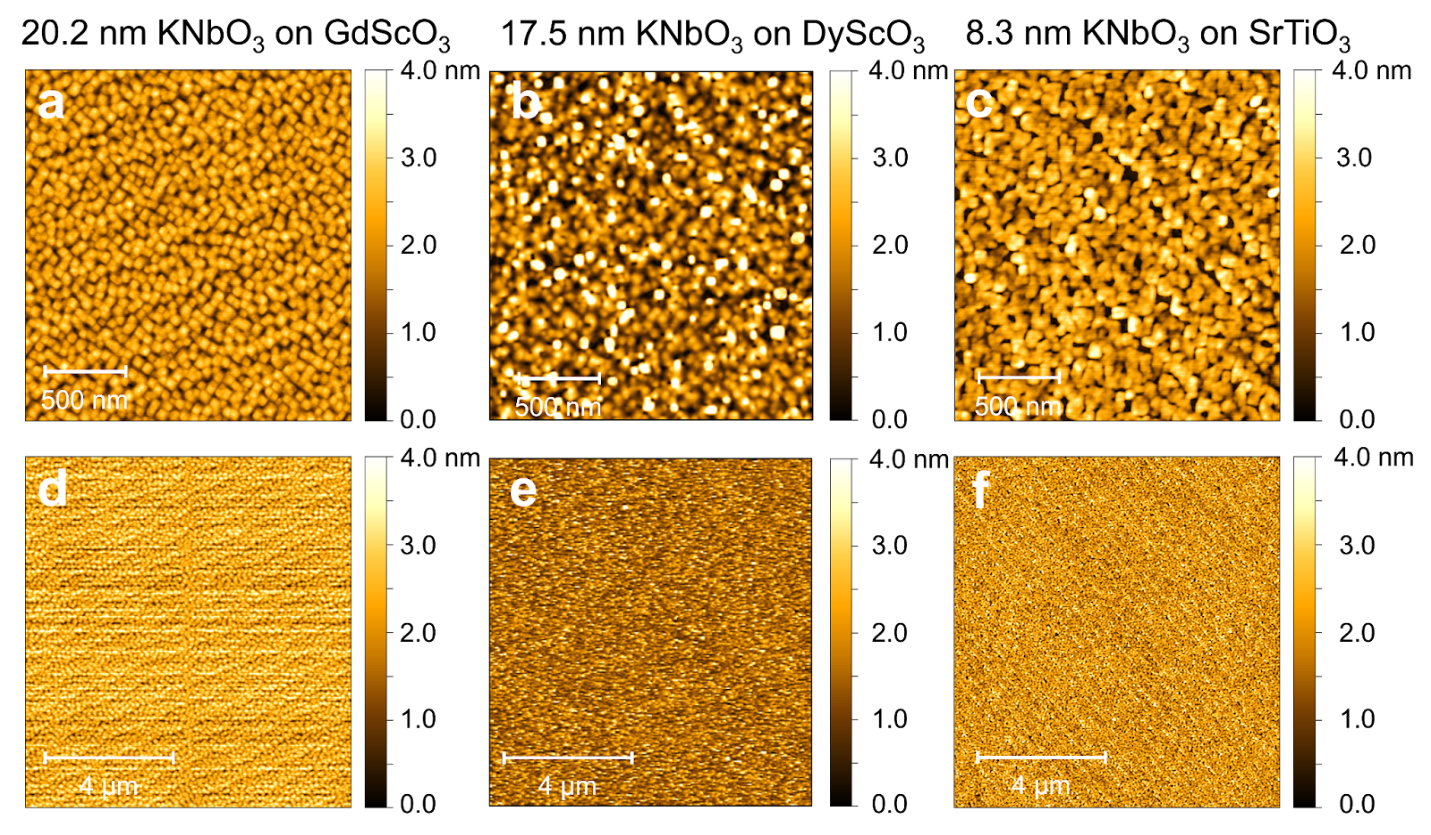


**Figure S5**: AFM images of KNbO_3_ films grown on **(a), (d) (**110)*_o_* GdScO_3_, (**b), (e)** (110)*_o_* DyScO_3_ and **(c), (f)** (001) SrTiO_3_ substrates, with RMS roughness ~ < 1 nm for all the three films.


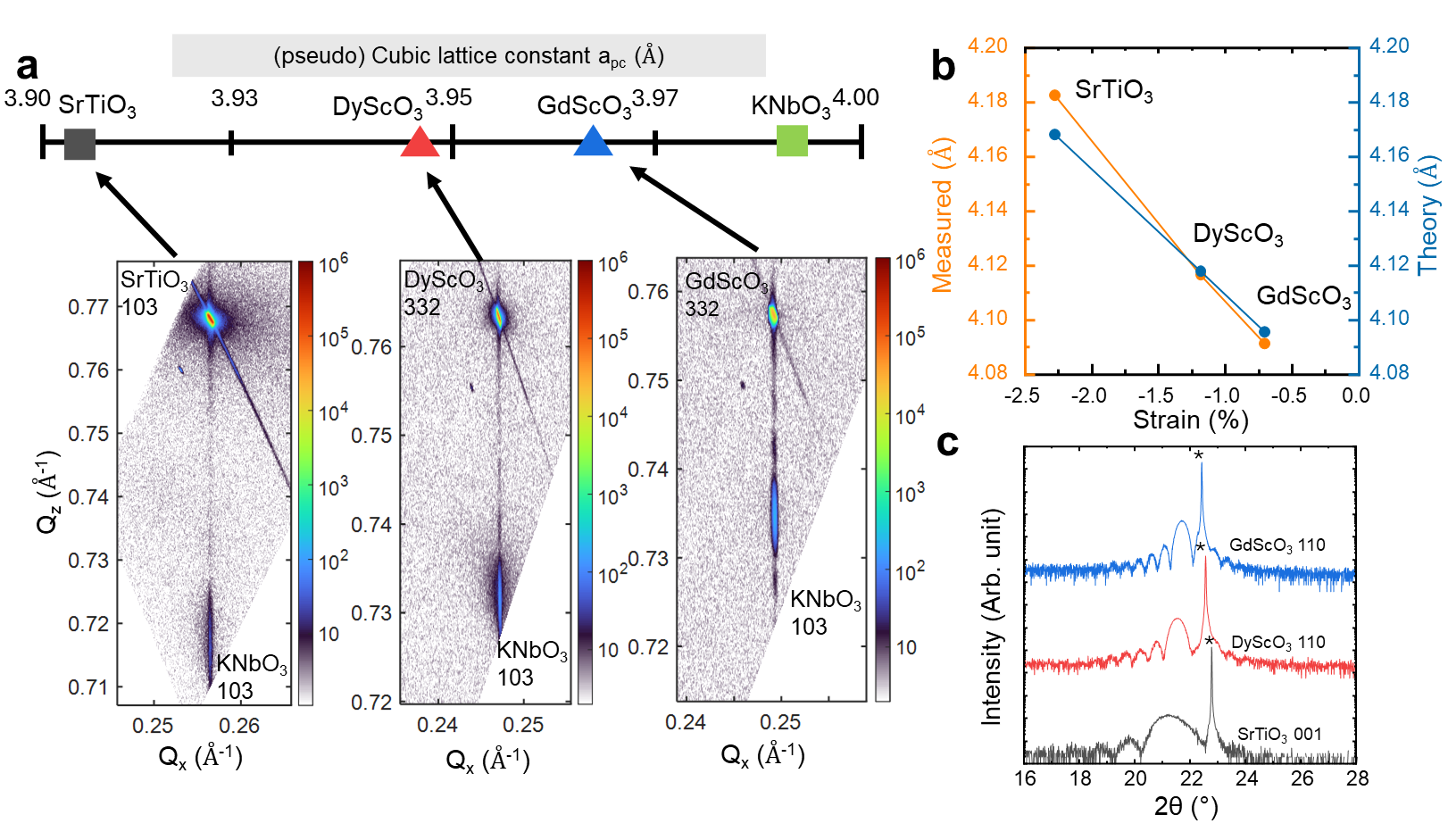


**Figure S6**: (a) Lab X-ray based reciprocal space maps for KNbO_3_ grown on SrTiO_3_, GdScO_3_ and SrTiO_3_ substrates showing coherent strained KNbO_3_ thin films on substrates. (b) Measured out-of-plane lattice parameter of KNbO_3_ as a function of strain measured by XRD, compared to expected lattice parameter from elastic theory. (c) $\theta-2\theta$XRD scans for the same KNbO_3_ films grown on various substrates, exhibiting Laue fringes indicating an abrupt interface between film and substrate. Only (00*l*) reflections are visible and were used to calculate the out-of-plane lattice constant in (b).


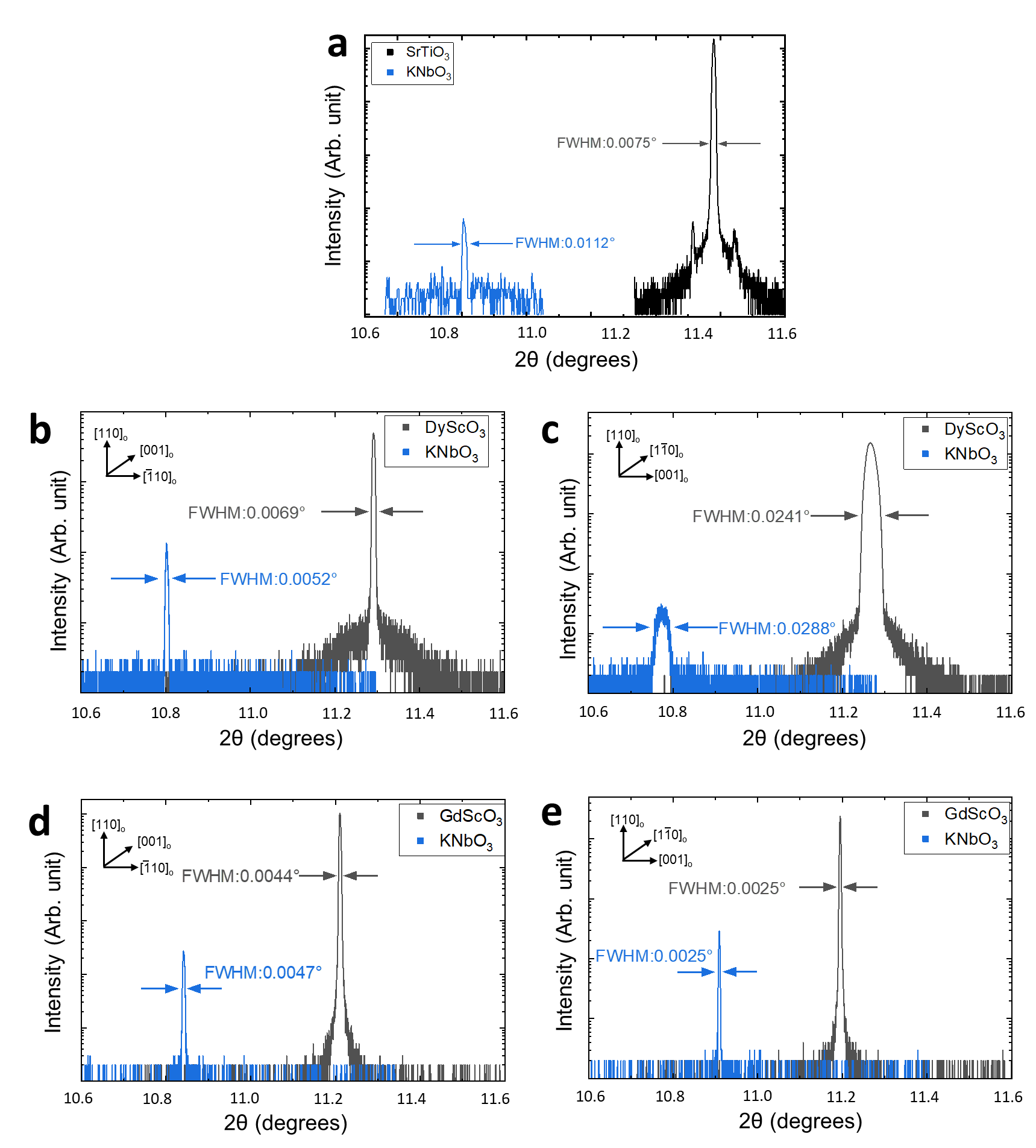


**Figure S7**: Lab X-ray based overlaid rocking curves for KNbO_3_ grown on SrTiO_3_, DyScO_3_ and GdScO_3_ substrates. (a) Overlaid 001 SrTiO_3_ and 001 KNbO_3_ peaks, showing comparable FWHMs. (b) and (c) Overlaid 001 KNbO_3_ and 110 DyScO_3_ peaks along the two orthogonal in-plane directions of the substrate. (b) and (c) Overlaid 001 KNbO_3_ and 110 GdScO_3_ peaks along the two orthogonal in-plane directions of the substrate.

**Note 5: Derivation of out-of-plane lattice constant calculations from elastic theory**

The out-of-plane lattice constant *a_⟂_* can be calculated from the out-of-plane strain, $\epsilon_{33}=\frac{\left( a_{\perp}-a_{KNO} \right)}{a_{KNO}}$ by expanding the tensor equation (in Einstein notation): $\sigma_{33}=c_{ijkl}\epsilon_{kl}$ for $\sigma_{33}$ and recognizing that $\sigma_{33}=0$ because the film is free of stress in the out-of-plane direction. This leads to:

$a_{\perp}=\frac{c_{KNO}\left( c_{33}2c_{13}\left( \frac{a_{STO}-a_{KNO}}{a_{KNO}} \right) \right)}{c_{33}}$ (1)

Where $c_{13}$ and $c_{33}$ are elastic stiffness tensor coefficients^[13]^ of KNbO_3_ in Voigt notation and $a_{STO}$and $a_{KNO}$ are the lattice constants of unstrained KNbO_3_ and SrTiO_3_, respectively. The calculated out-of-plane lattice constant expected for a commensurately strained KNbO3 film on SrTiO_3_ at room temperature is 4.168 Å (**Figure S6b**). This is lower than the 4.182 Å ± 0.015 Å value measured by X-ray diffraction for the commensurately strained 8.3 nm thick KNbO_3_ films.

In contrast to the extended out-of-plane lattice spacing observed for the commensurately strained KNbO_3_ film grown on a SrTiO_3_ substrate, the 17.5 and 20.2 nm thick commensurately strained KNbO_3_/DyScO_3_ and KNbO_3_/GdScO_3_ shown in **Figure S6 b - c** agree with the expected out-of-plane spacing, calculated with elastic theory. Because GdScO_3_ is orthorhombic, the in-plane biaxial strains $\epsilon_{11}$ and $\epsilon_{22}$ imposed by the substrate are no longer equal and the equation for *a_⟂_* becomes,

$$a_{\perp}=-\frac{c_{13}c_{KNO}\left( a_{GSO_{170}}-4a_{KNO}+a_{GSO_{001}} \right)}{2a_{KNO}c_{33}}$$

where $a_{GSO001}$ and $a_{GSO110}$ are the in-plane distances that establish _11_ and _22_ through commensurate strain. Specifically, $a_{GSO001}$ is the c-axis length of GdScO_3_ (7.931 Å) and $a_{GSO110}$is the 110 of GdScO_3_ (7.940 Å) and $a_{DSO001}$ is the c-axis length of DyScO_3_ (7.903 Å) and $a_{DSO110}$ is the 110 of DyScO_3_ (7.891 Å)^[14]^ where we are using the non-standard *Pbnm* setting of GdScO_3_ and DyScO_3_ as is most common in the literature. Here the calculations result in an expected spacing for GdScO_3_ of 4.095 Å and 4.118 Å for DyScO_3_ at room temperature compared to the 4.091 Å ± 0.015 Å for GdScO_3_ and 4.117 Å ± 0.015 Å for DyScO_3_ measured by X-ray diffraction. The films grown on GdScO_3_and DyScO_3_ do not show a large deviation between the measured and calculated out-of-plane lattice parameters.

**Note 6: Transmission Electron Microscopy**


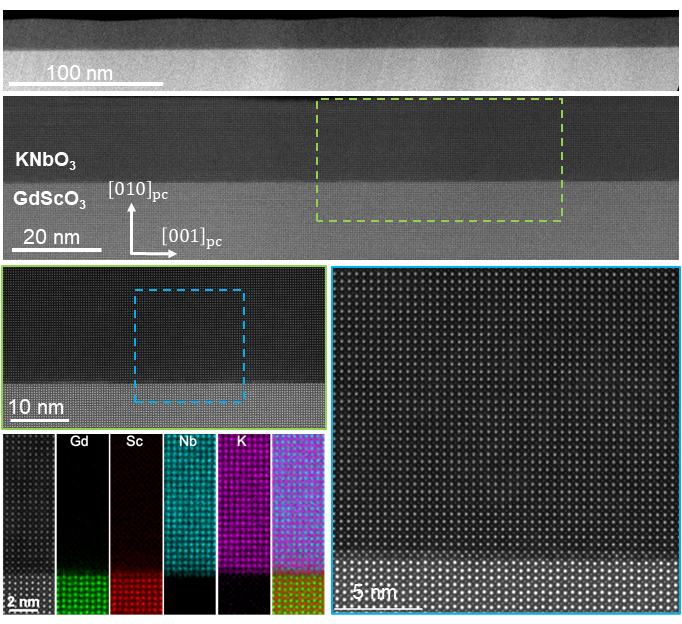


**Figure S8:** HAADF-STEM images of KNbO_3_ thin film grown on GdScO_3_ substrate. The interface structure is uniform throughout the sample, indicating a coherent strain between the film and the substrate. No extended defects are observed, which suggests high-quality epitaxial growth. The atomic resolution EDX map reveals an intermixing of metal ions at the interface, primarily involving scandium from the substrate and niobium from the film. This intermixed layer is crucial for maintaining charge neutrality at the KO terminating layer. The absence of defects and the coherent strain between the KNbO_3_ film and the GdScO_3_ substrate underscore the high quality of the epitaxial growth.

**
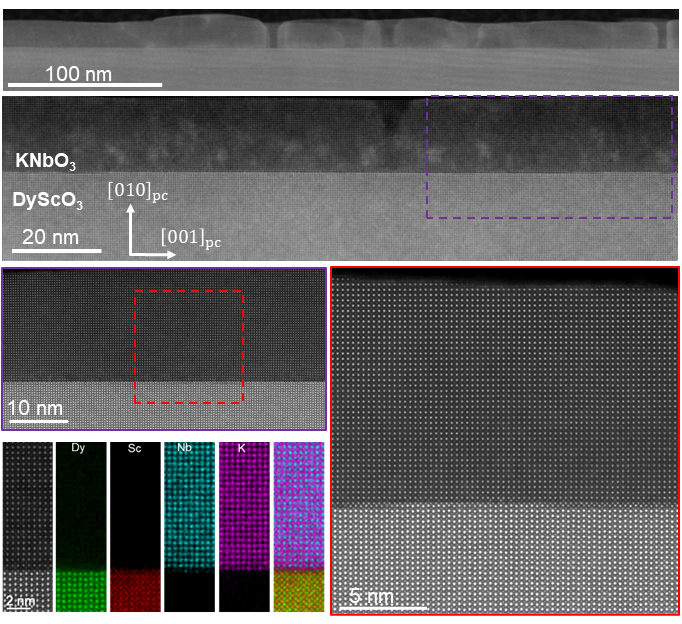
**

**Figure S9:** HAADF-STEM images of KNbO_3_ thin film grown on DyScO_3_ substrate. At low magnification, the growth appears to follow the island growth model rather than a layer-by-layer growth, as evidenced by the presence of islands throughout the TEM sample. Additionally, low-angle grain boundaries are observed within the film, suggesting some degree of misorientation between the grains. High-magnification images, taken from the larger grains, show that the interface between the film and the substrate is uniform and that the film is coherently strained to the substrate in these regions. This coherence indicates that, despite the island growth mode, large grains maintain a high-quality interface. The atomic resolution EDX map reveals similar intermixing of metal ions at the interface as seen for KNbO_3_ film on GdScO_3_ substrate. This intermixed layer is essential for maintaining the charge neutrality of the KO terminating layer, similar to the behavior observed in the KNbO_3_/GdScO_3_ system.

**
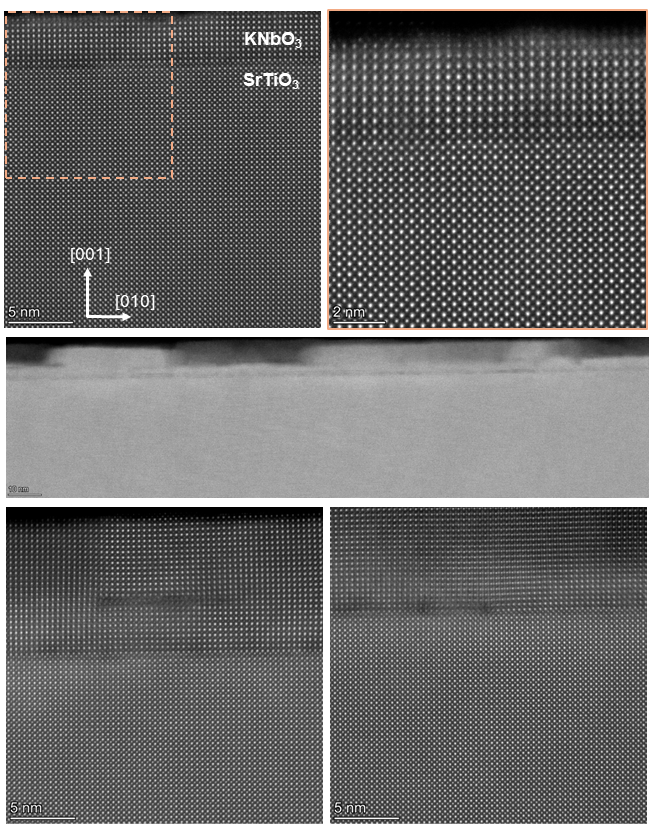
**

**Figure S10:** HAADF-STEM images of KNbO_3_ thin film grown on SrTiO_3_ substrate. The high-magnification images reveal small regions where the film has grown uniformly and without defects on the substrate, indicating localized areas of successful epitaxial growth. However, the larger area images indicate that both the interface and the film contain a high density of defects. The significant lattice mismatch between film and SrTiO_3_ contributes to the presence of these defects. This large mismatch induces strain in the film, leading to dislocations, misfit dislocations, and other defects as the film attempts to relieve the strain. The high density of these defects disrupts the overall uniformity and coherence of the film. Due to the high density of defects, the film did not withstand the electron dose required for atomic resolution EDX mapping. The instability of the defective regions under the electron beam led to rapid degradation, preventing detailed chemical analysis. This behavior is typical in materials with high defect densities, where the structure is less robust under high-dose electron irradiation.

**
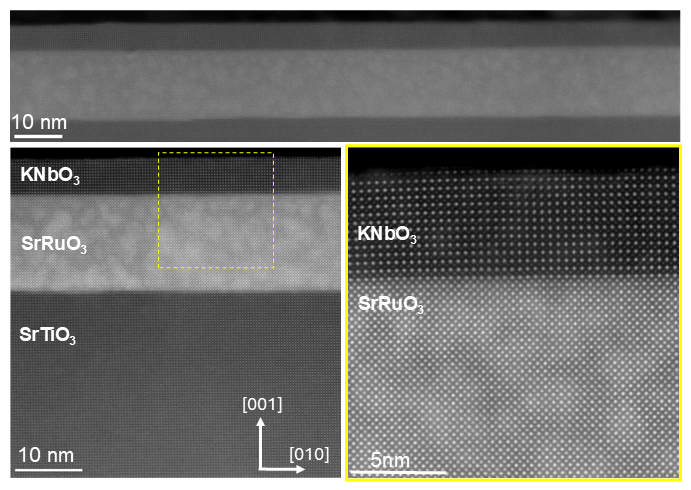
**

**Figure S11:** HAADF-STEM images of KNbO_3_ thin film grown on SrTiO_3_ substrate with a SrRuO_3_ bottom electrode. Unlike KNbO_3_ grown directly on SrTiO_3_ substrate, with the introduction of a SrRuO_3_ bottom electrode (15 nm thick) no extended defects are observed in the KNbO_3_ film, which suggests high-quality epitaxial growth. The interface structure is uniform throughout the sample, indicating a coherent strain between the KNbO_3_ film, SrRuO_3_ bottom electrode and SrTiO_3_ substrate. This suggests significant improvement in KNbO_3_ thin film quality with the introduction of a SrRuO_3_ bottom electrode.

**Note 7: X-ray photoelectron spectroscopy (XPS)**

**
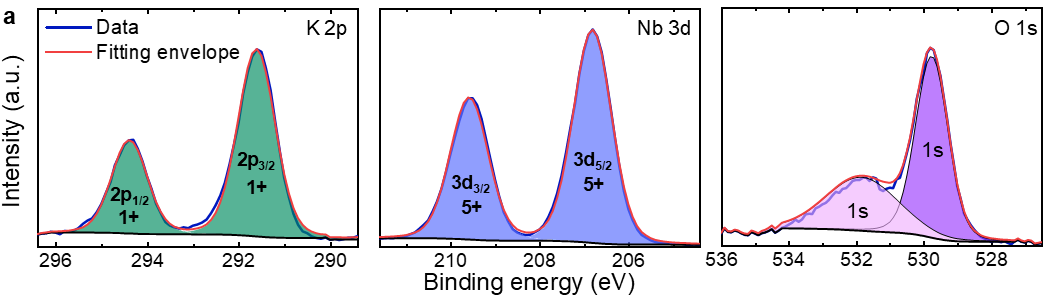
**

**Figure S12:** XPS spectra of KNbO3 thin film done on KNbO_3_/SrRuO_3_/SrTiO_3_ sample corresponding to **(a)** K 2p **(b)** Nb 3d and **(c)** O 1s core levels.

X-ray photoelectron spectroscopy (XPS) was done on KNbO_3_/SrRuO_3_/SrTiO_3_ to confirm the valence state of the elements in KNbO_3_ films. The spectra show two peaks at 291.6 eV and 294.3 eV respectively corresponding to K 2p_3/2_ and K 2p_1/2_ respectively confirming the +1 oxidation of K^[15,16]^. The Nb peaks corresponding to Nb 3d_5/2_ and Nb 3d_3/2_ levels are observed at 206.7 eV and 209.4 eV which are associated with +5 oxidation state of Nb^[15,17]^. The O 1s peak at 529.8 eV is attributed to -2 oxidation state of O. A smaller shoulder peak is also observed at 531.7 eV which is associated to surface bound hydroxyl groups (-OH)^[15]^.

**Note 8: Derivation of Optical Second Harmonic Generation (SHG) polarimetry expressions for the tetragonal and monoclinic phases**

A schematic of the SHG setup is shown in **Figure 2a** of the main text. The lab coordinates (X, Y, Z) attached to the direction of the incoming beam: X || *p* polarization, Y || *s* polarization, where *p* and *s* are defined by a rotating half wave plate as labelled in **Figure 2a**. The crystal axes coordinate *i =* (1,2,3) are attached to the sample and related to the substrate as follows:

For GdScO_3_ and DyScO_3_ substrates: 1 || [001]_o_ , 2 || [1-10]_o_ , 3 || [110]_o_ , where subscript “o” refers to orthorhombic unit cell of the scandate substrates.

For SrTiO_3_ substrate: 1 || [100], 2 || [010], 3 || [001]_._

In lab coordinates (X, Y, Z), the electric field of the incident beam can be written as $(E_{o}\cos\varphi, E_{o}sin \varphi, 0)$ where *φ* is the polarization rotation angle introduced by the half wave plate as shown in **Figure 2a**. For an incidence angle *ϴ* on the sample (see **Figure 2a**), the electric field in the crystal axes coordinates can be expressed as $\left( E_{o}\cos\varphi\cos\theta,E_{o}\sin\varphi,-E_{o}\cos\varphi\sin\varphi) \right.$. The induced nonlinear polarization *P^2ω^*, is related to the incident electric field through the nonlinear susceptibility tensor, *d_ijk_* through the following equation:

$P_{i}^{2\omega}\propto d_{ijk}E_{j}^{\omega}E_{k}^{\omega}$ (1)

The proportionality constants depend on incident beam fluence, Fresnel’s coefficients at the film-air and film-substrate interfaces and the thickness of the films.

**Tetragonal model**:

For tetragonal unit cell the nonlinear susceptibility can be written in Voigt notation as:

$$\left( \begin{matrix} 0 & 0 & 0 & 0 & d_{15} & 0 \\ 0 & 0 & 0 & d_{15} & 0 & 0 \\ d_{31} & d_{31} & d_{33} & 0 & 0 & 0 \end{matrix} \right)$$

The induced nonlinear polarization in the crystal coordinates *i =* (1,2,3) (calculated through equation 1) can be rotated back to lab coordinates (X, Y, Z), to give the *p* and *s*-polarized components of the SHG (*p* || X and *s* || Y). The *p* and *s*-polarized SHG intensities can be expressed as follows:

For normal incidence ($\theta=0^{\circ}$)

$I_{p}^{2\omega}\propto\left( P_{p}^{2\omega} \right)^{2}=0$

$$I_{s}^{2\omega}\propto\left( P_{s}^{2\omega} \right)^{2}=0$$

For oblique incidence $(\theta=45^{\circ})$

$I_{p}^{2\omega}\propto\left( P_{p}^{2\omega} \right)^{2}\propto\left( (2d_{15}-d_{31}-d_{33})cos[\varphi]^{2}-2d_{31}sin[\varphi]^{2} \right)^{2}$ (2)

$$I_{s}^{2\omega}\propto\left( P_{s}^{2\omega} \right)^{2}\propto d_{15}^{2}Sin[2\varphi]^{2}$$

**Figure S13** and upper panel of main text **Figure 2b** show the tetragonal model fitting for KNbO_3_ on SrTiO_3_ (10K and 300K), KNbO_3_ on DyScO_3_ (300K) and KNbO_3_ on GdScO_3_ (300K)

**Monoclinic model**:

For the low temperature monoclinic phase (*M_c_*) in KNbO_3,_ a multi-domain model is assumed consistent with X-ray measurements and phase field theory simulations. In crystal physics coordinates *i =* (1, 2, 3), nonlinear susceptibility in the monoclinic phase can be written as:

$$d_{ij}=\left( \begin{matrix} d_{11} & d_{12} & d_{13} & 0 & d_{15} & 0 \\ 0 & 0 & 0 & d_{24} & 0 & d_{26} \\ d_{31} & d_{32} & d_{33} & 0 & d_{35} & 0 \end{matrix} \right)$$

Here, the monoclinic mirror plane is perpendicular to the crystallographic *b*-axis of the monoclinic cell of KNbO_3_ which is parallel to *i*=2 crystal physics coordinate. Through rotating the unit cell by right angles, 4 such unit cells can be achieved, each associated with an area fraction labelled as below:

Domain 1: *a* || 1, *b* || 2, *c* || 3 (Area fraction: A_1_)

Domain 2: *a* || 2, *b* || -1, *c* || 3 (Area fraction: A_2_)

Domain 3: *a* || -1, *b* || -2, *c* || 3 (Area fraction: A_3_)

Domain 4: *a* || -2, *b* || 1, *c* || 3 (Area fraction: A_4_)

Here (*a*, *b*, *c*) denote the crystallographic axes of the monoclinic unit cell, *i =* (1,2,3) denote the previously defined crystal physics coordinate system and the area fractions are constrained to A_1_ + A_2_+ A_3_ + A_4_ = 1.

For normal incidence ($\theta=0^{\circ}$)

From Equation 1, the induced nonlinear polarization can be calculated in the crystal coordinate system and can be transformed into lab coordinates (X, Y, Z). The resultant nonlinear polarization in the lab coordinates X (*p*-polarized) and Y (*s*-polarized) are tabulated below (Table S5):

**Table S5: *p* and *s*-polarization components for different domains**

|  | $P_{p}^{2\omega}$ | $P_{s}^{2\omega}$ |
| --- | --- | --- |
| Domain 1 | $d_{11}{cos}^{2}\varphi+d_{12}{sin}^{2}\varphi$ | $d_{26}sin2\varphi$ |
| Domain 2 | $d_{26}sin2\varphi$ | $d_{12}{cos}^{2}\varphi+d_{11}{sin}^{2}\varphi$ |
| Domain 3 | ${-(d}_{11}{cos}^{2}\varphi+d_{12}{sin}^{2}\varphi)$ | $-d_{26}sin2\varphi$ |
| Domain 4 | $-d_{26}sin2\varphi$ | ${-(d}_{12}{cos}^{2}\varphi+d_{11}{sin}^{2}\varphi)$ |

The effective SHG intensity can be calculated as follows:

$$I_{p}^{2\omega}\propto\left( P_{p}^{2\omega} \right)^{2}\propto\left( A_{1}P_{p, domain 1}^{2\omega}+A_{2}P_{p, domain 2}^{2\omega}+A_{3}P_{p, domain 3}^{2\omega}+A_{4}P_{p, domain 4}^{2\omega} \right)^{2}$$

This can be simplified to:

$I_{p}^{2\omega}\propto K_{1p}\left( {sin}^{2}\varphi+K_{2p}{cos}^{2}\varphi\right)^{2}+K_{3p}{sin}^{2}2\varphi+K_{4p}\left( {sin}^{2}\varphi+K_{2p}{cos}^{2}\varphi\right)sin2\varphi$ (3)

where,

$$K_{1p}=\delta A_{1}^{2}d_{12}^{2} K_{2p}=\frac{d_{11}}{d_{12}} K_{3p}=\delta A_{2}^{2}d_{26}^{2} K_{4p}=2\delta A_{1}\delta A_{2}d_{12}d_{26}$$

$\delta A_{1} = A_{1} - A_{3}$and $\delta A_{2} = A_{2} - A_{4}$. Similar expressions can also be derived for $I_{s}^{2\omega}$ with the following coefficients:

$$K_{1s}=\delta A_{2}^{2}d_{11}^{2} K_{2s}=\frac{d_{12}}{d_{11}} K_{3s}=\delta A_{1}^{2}d_{26}^{2} K_{4s}=2\delta A_{1}\delta A_{2}d_{11}d_{26}$$

Similar expression has also been shown earlier for multidomain *mm*2 model in epitaxial thin films ^[18,19]^. **Figure S14** shows the normal incidence polarimetry for KNbO_3_ on GdScO_3_ and DyScO_3_ at 10 K fitted to a multidomain monoclinic model.

For oblique incidence ($\theta=45^{\circ}$):

Equations for oblique incidence polarimetry involve more convoluted combinations of *d*-coefficients and area fractions, however they can be reduced to the same form as equation (3). The exact convolutions are listed below:

$$\begin{aligned} &K_{1p}=\frac{1}{8}\left[ 2\left( A_{1}+A_{3} \right)d_{32}+2\delta A_{1}d_{12}+2d_{31}(A_{2}+A_{4}) \right]^{2} \end{aligned}$$

$$\begin{aligned} K_{2p}=\frac{1}{2\sqrt{2K_{1p}}}\left[ \delta A_{1}\left( d_{11}+d_{13}-2d_{35} \right)+\left( A_{1}+A_{3} \right)\left( d_{31}+d_{33}-2d_{15} \right) \right.+\left( A_{2}+A_{4} \right)\left( d_{32}+d_{33}-2d_{24} \right] \end{aligned}$$

$$K_{3p}=\frac{\left[ \delta A_{2}\left( d_{26}-d_{35} \right)]^{2} \right.}{4}$$

$$K_{4p}=2\sqrt{K_{1p}K_{3p}}$$

$$K_{1s}=\frac{1}{4}\left( A_{2}-A_{4} \right)^{2}d_{11}^{2}$$

$$K_{2s}=\frac{1}{2\sqrt{K_{1s}}}\left( d_{12}+d_{13} \right)\left( A_{2}-A_{4} \right)$$

$$K_{3s}=\frac{1}{4}\left( \sqrt{2}A_{1}\left( d_{26}-d_{24} \right)-\sqrt{2}A_{3}\left( d_{26}+d_{24} \right)+\frac{d_{15}}{\sqrt{2}}\left( A_{4}-A_{2} \right) \right)^{2}$$

$$K_{4s}=2\sqrt{K_{1s}K_{3s}}$$

**
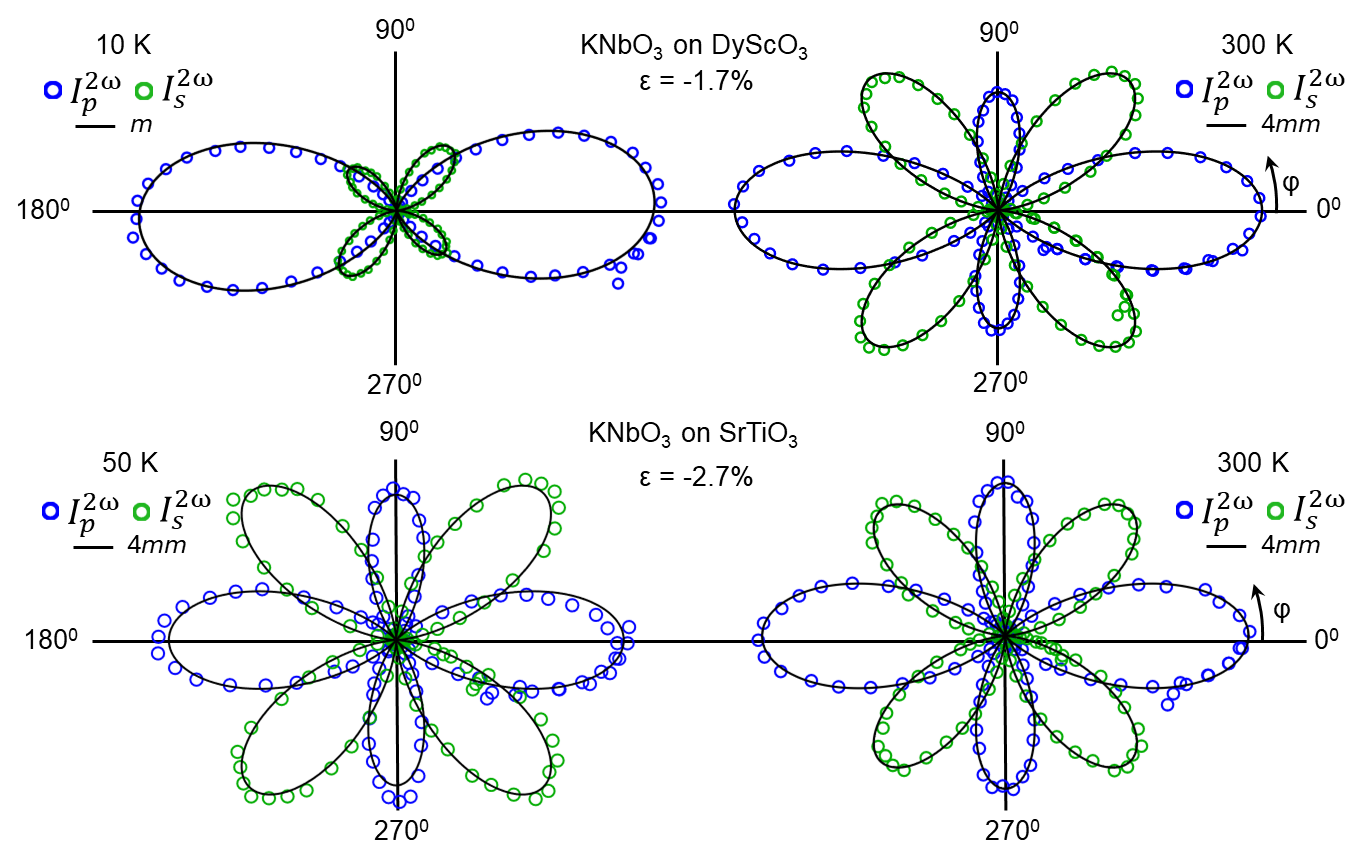
**

**Figure S13:** Oblique-incidence SHG polarimetry at T = 300 K and T = 10 K for KNbO_3_ film on DyScO_3_ and SrTiO_3_ substrates showing a tetragonal model fitting for both KNbO_3_ on DyScO_3_ and SrTiO_3_ at 300 K. While at 10 K, a multidomain monoclinic model is necessary for KNbO_3_ on DyScO_3_ but KNbO_3_ on SrTiO_3_ can still be fitted with a tetragonal model similar to 300 K.

**
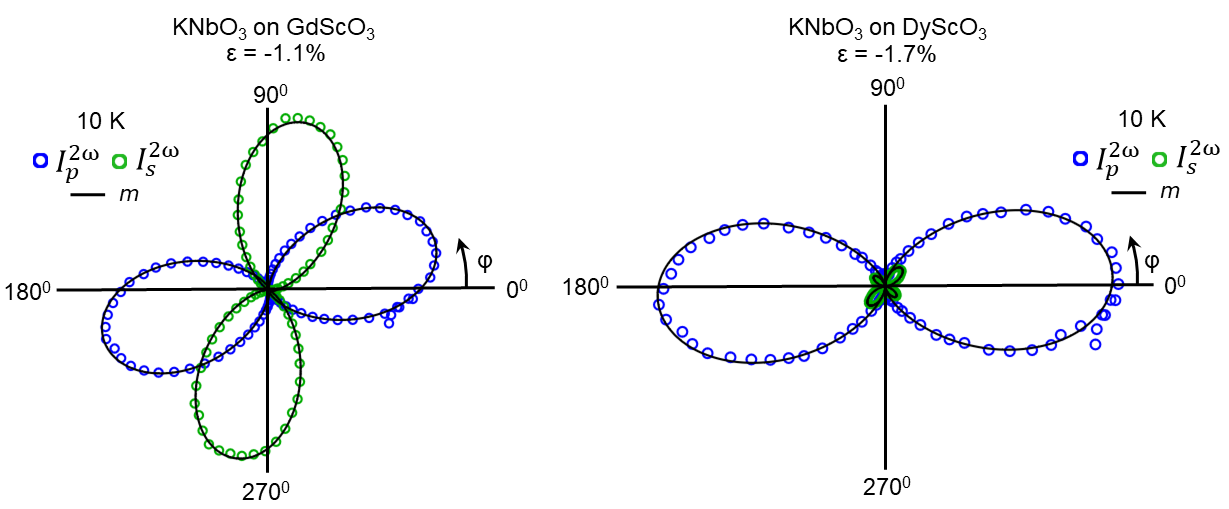
**

**Figure: S14:** Normal incidence SHG polarimetry at T = 10 K for KNbO_3_ on DyScO_3_ and GdScO_3_ fitted to multdomain monoclinic model.

**Note 9: High temperature decomposition of KNbO_3_ on GdScO_3_ film measured by SHG**

**
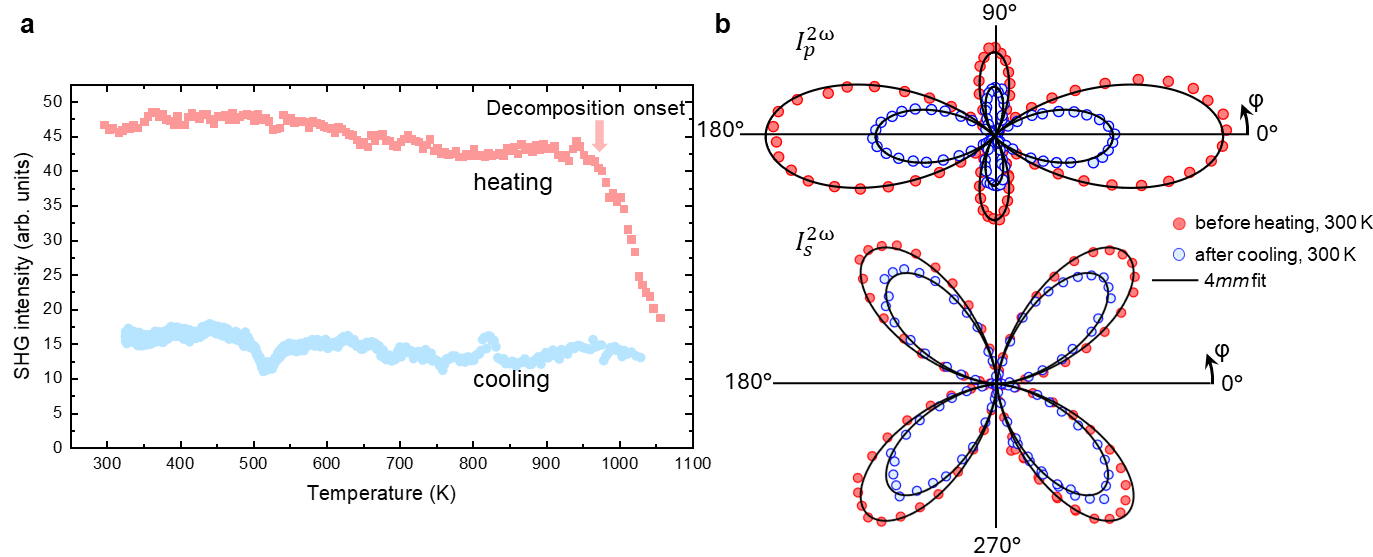
**

**Figure S15**: a) SHG intensity vs temperature in the high temperature regime for KNbO_3_ on GdScO_3_. On heating the sample shows irreversible degradation around 975 K, while cooling run shows steady SHG intensity down to room temperature. b) SHG polarimetry at room temperature before starting heating compared to after cooling back down for *p*-polarized (upper panel) and *s*-polarized (lower panel) SHG. SHG polarimetry before heating and after cooling down can both be fitted to a 4*mm* model showing the same symmetry, indicating that the only change occurring is in the intensity.

**Note 10: Calculation of Nonlinear optical coefficients in tetragonal and monoclinic symmetry**

**Room temperature tetragonal:**

#*SHAARP.ml*^[20]^ package was used characterize the nonlinear optical coefficients of KNbO_3_ thin films on different substrates. The measurement geometry is shown in **Figure 2a** of the main text.

The linear optical properties were first characterized via ellipsometry, and x-ray reflectivity was used to measure the thickness of the films. These values are tabulated below in **Table S6** for both the film and substrate, which also serve as the input parameters for the #*SHAARP.ml* package.

| Layer | *n* (400 nm) | *n* (800 nm) | Thickness |
| --- | --- | --- | --- |
| KNbO_3_ | 2.565 + 0.107 *i* | 2.260 + 0.110 *i* | 20.2 nm |
| GdScO_3_ | 2.090 + 0.049 *i* | 2.021 + 0.023 *i* | Substrate |

| Layer | *n* (400 nm) | *n* (800 nm) | Thickness |
| --- | --- | --- | --- |
| KNbO_3_ | 2.610 + 0.067 *i* | 2.308 + 0.029 *i* | 17.5 nm |
| DyScO_3_ | 2.084 + 0.068 *i* | 2.016 + 0.032 *i* | Substrate |

| Layer | *n* (400 nm) | *n* (800 nm) | Thickness |
| --- | --- | --- | --- |
| KNbO_3_ | 2.419 + 0.046 *i* | 2.163 + 0.006 *i* | 8.5 nm |
| SrTiO_3_ | 2.678 + 0.064 *i* | 2.350 + 0.021 *i* | Substrate |

**Table S6:** Complex refractive index coefficients and thickness values of KNbO_3_ thin films on GdScO_3_, DyScO_3_ and SrTiO_3_ substrates.

Equations for SHG generated for KNbO_3_ thin films on the different substrates are generated through the full multiple reflection assumptions in the #*SHAARP.ml* package, which considers multiple reflections in the two layers (thin film + substrate) for both the fundamental and second harmonic beams. By calibrating the SHG generated from the thin films to a standard LiNbO_3_ reference sample, full characterization of the SHG tensor is possible.

For tetragonal unit cell the SHG tensor can be written in Voigt notation as:

$$\left( \begin{matrix} 0 & 0 & 0 & 0 & d_{15} & 0 \\ 0 & 0 & 0 & d_{15} & 0 & 0 \\ d_{31} & d_{31} & d_{33} & 0 & 0 & 0 \end{matrix} \right)$$

Simultaneous fitting of p-polarized generated SHG for 30°, 45°, 60° incidence angle gives the $d_{33}/d_{15}$ and $d_{31}/d_{15}$ ratios. The *s*-polarized SHG generated from the sample depends only on the $d_{15}$ coefficient and can be calibrated to LiNbO_3_.

The general form of *p* and *s*-polarized SHG for any angle and sample can be written as:

$$I_{p}^{2\omega}\propto\left[ \left( A_{1p}d_{15}+A_{2p}d_{31}+A_{3p}d_{33} \right)\cos[\varphi]^{2}+A_{4p}d_{31}\sin[\varphi]^{2} \right]\times[cc]$$

$$I_{s}^{2\omega}\propto[{A_{1s}d}_{15}\mathrm{Sin}[2\varphi]]\times[cc]$$

The coefficients are detailed in the following table corresponding to each sample and measurement angle.

| Film | Angle | $A_{1p}$ | $A_{2p}$ | $A_{3p}$ | $A_{4p}$ | $A_{1s}$ |
| --- | --- | --- | --- | --- | --- | --- |
| KNbO_3_ on GdScO_3_ | 30° | $-5.7+9.6 i$ | $1.7 - 3.8 i$ | $0.1 - 0.2 i$ | $1.7 - 3.7 i$ |  |
|  | 45° | $12.2 - 14.4 i$ | $-3.9 + 6.1 i$ | $-0.3 + 0.7 i$ | $-3.6 + 5.4 i$ | $-11.1 - 12.1 i$ |
|  | 60° | $7.7 - 14.9 i$ | $-2.3 +5.9 i$ | $-0.2 + 1.0 i$ | $-2.0 +4.5 i$ |  |
| KNbO_3_ on DyScO_3_ | 30° | $-8.4+9.1 i$ | $2.5 - 3.8 i$ | $0.1 - 0.1 i$ | $2.5 - 3.7 i$ |  |
|  | 45° | $11.0 - 12.8 i$ | $-3.3 + 5.2 i$ | $-0.3 + 0.5 i$ | $-3.2 + 4.8 i$ | $-10.3 - 10.8 i$ |
|  | 60° | $11.5 - 14.6 i$ | $-3.5 + 5.9 i$ | $-0.5 + 0.9 i$ | $-3.0 +4.6 i$ |  |
| KNbO_3_ on SrTiO_3_ | 30° | $-1.7+6.5 i$ | $0.9 - 3.1 i$ | $0.0 - 0.1 i$ | $0.8 - 3.0 i$ |  |
|  | 45° | $2.3 - 9.0 i$ | $-1.2 + 4.3 i$ | $-0.1 + 0.5 i$ | $-1.0 + 3.8 i$ | $-0.3 - 2.5 i$ |
|  | 60° | $2.7 - 10.1 i$ | $-1.3 + 4.8 i$ | $-0.2 + 0.9 i$ | $-0.9 + 3.5 i$ |  |

**Table S7:** $A_{1p}$, $A_{2p}$, $A_{3p}$, $A_{4p}$ and $A_{1s}$ coefficients corresponding to all three strained films corresponding to $I_{p}^{2\omega}$ and $I_{s}^{2\omega}$ equations.

The resulting SHG coefficients are tabulated below:

| Film | $d_{15}$ (pm/V) | $d_{31}$(pm/V) | $d_{33}$ (pm/V) |
| --- | --- | --- | --- |
| KNbO_3_ on SrTiO_3_ | $49\pm1$ | $44\pm1$ | $59\pm1$ |
| KNbO_3_ on DyScO_3_ | $37\pm1$ | $31\pm1$ | $41\pm1$ |
| KNbO_3_ on GdScO_3_ | $32\pm1$ | $32\pm1$ | $51\pm1$ |

**Table S8:** $d_{15}$, $d_{31}$, $d_{33}$ SHG tensor coefficients of room temperature tetragonal phase of KNbO_3_ corresponding to all three strained films.

**Note 11: Temperature dependent reciprocal space mapping of KNbO_3_ on GdScO_3_**


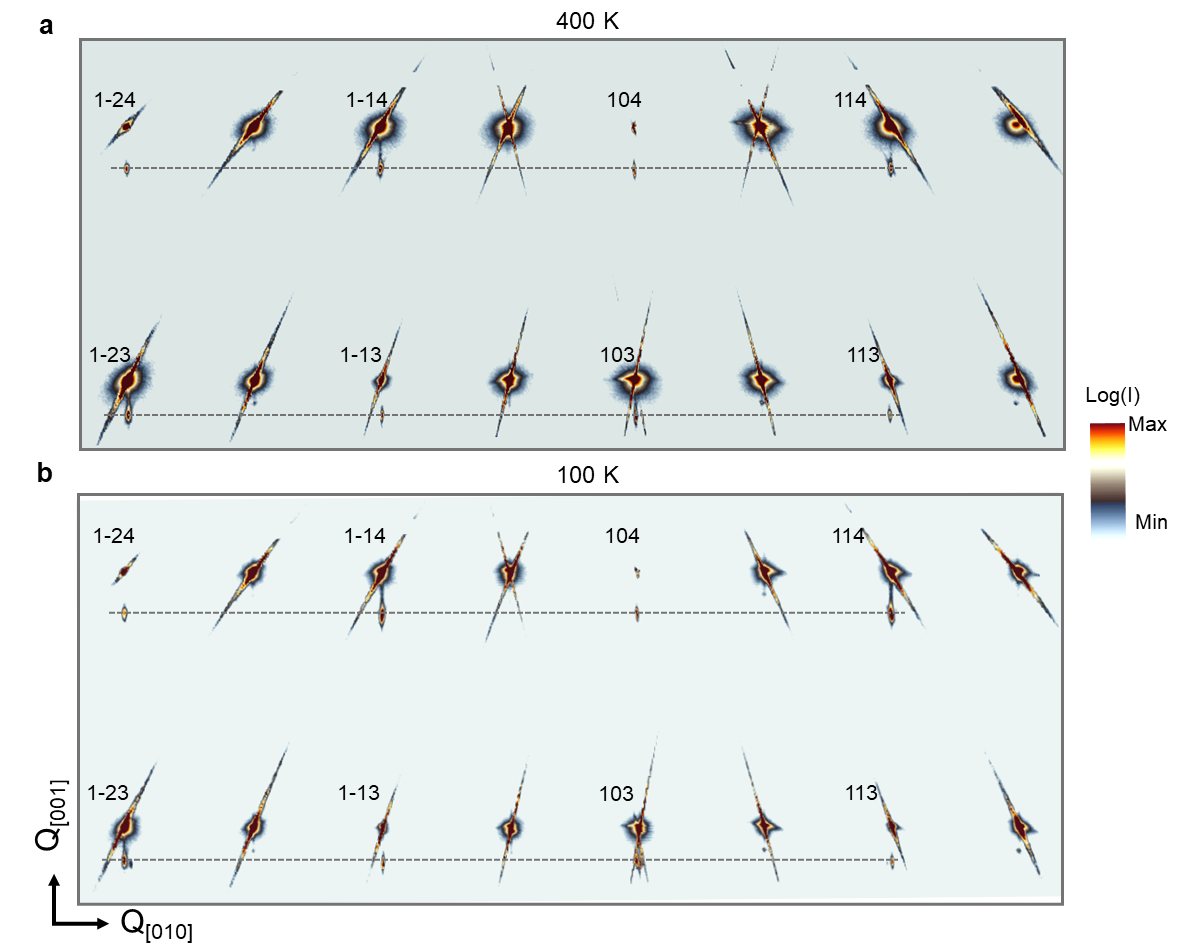


**Figure S16:** Reciprocal space maps (RSM) capturing the effect of temperature on KNbO_3_ film structures relative to the GdScO_3_ substrate. The maps at 400 K (a) and 100 K (b) correspond to temperatures above and below the tetragonal to monoclinic phase transition (275 K) respectively. The pseudocubic Miller indices of several off-specular substrate peaks are marked in inset. Horizontal dotted lines mark the position of the film peaks relative to each other and the substrate peaks. The film peak alignment along the horizontal [010]-direction indicates that the peak shifts relative to the substrate are only dependent on $Q_{001}$ and do not depend on the Miller indices of the substrate. This supports strain as the dominating factor for peak position – signifying the average tetragonal structure of the film at both 100 K and 400 K, while the additional occurrence (See **Figure 3b**) of the tilted satellite peaks at 100 K along the <110> direction signify the monoclinic distortion at low temperature.

**
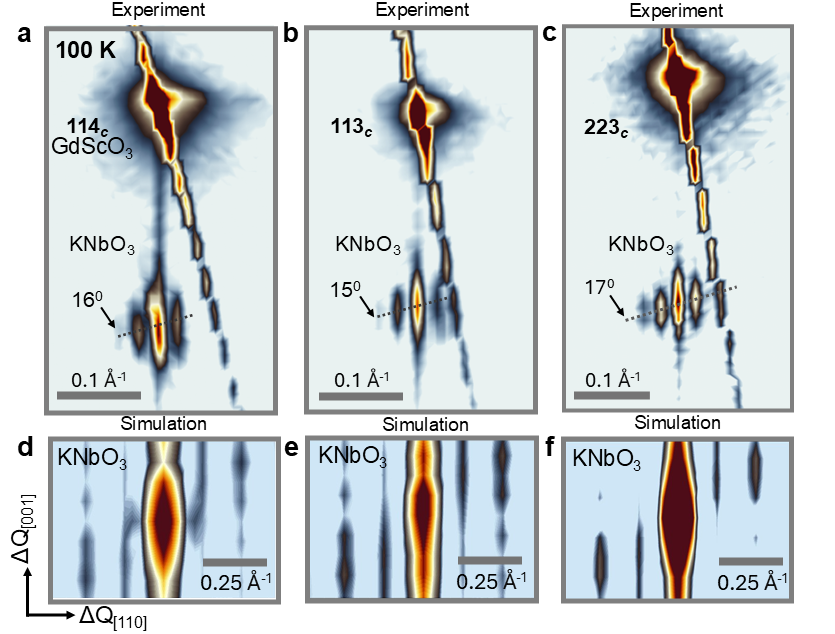
**

**Figure S17:** Experimentally measured RSM images around different GdScO_3_ Bragg peaks (**a**: 114*_c_*, **b**: 113*_c_*, **c**: 223*_c_*) at 100 K showing the tilt of diffused satellites due to tilted monoclinic domain walls. Diffraction simulations conducted on phase-field simulated microstructure at 100 K showing tilted diffused satellite peaks around corresponding KNbO_3_ peaks measured experimentally (**d**: 114*_c_*, **e**: 113*_c_*, **f**: 223*_c_*).

**Note 12: Structural characterization of KNbO_3_ thin films with SrRuO_3_ bottom electrodes**


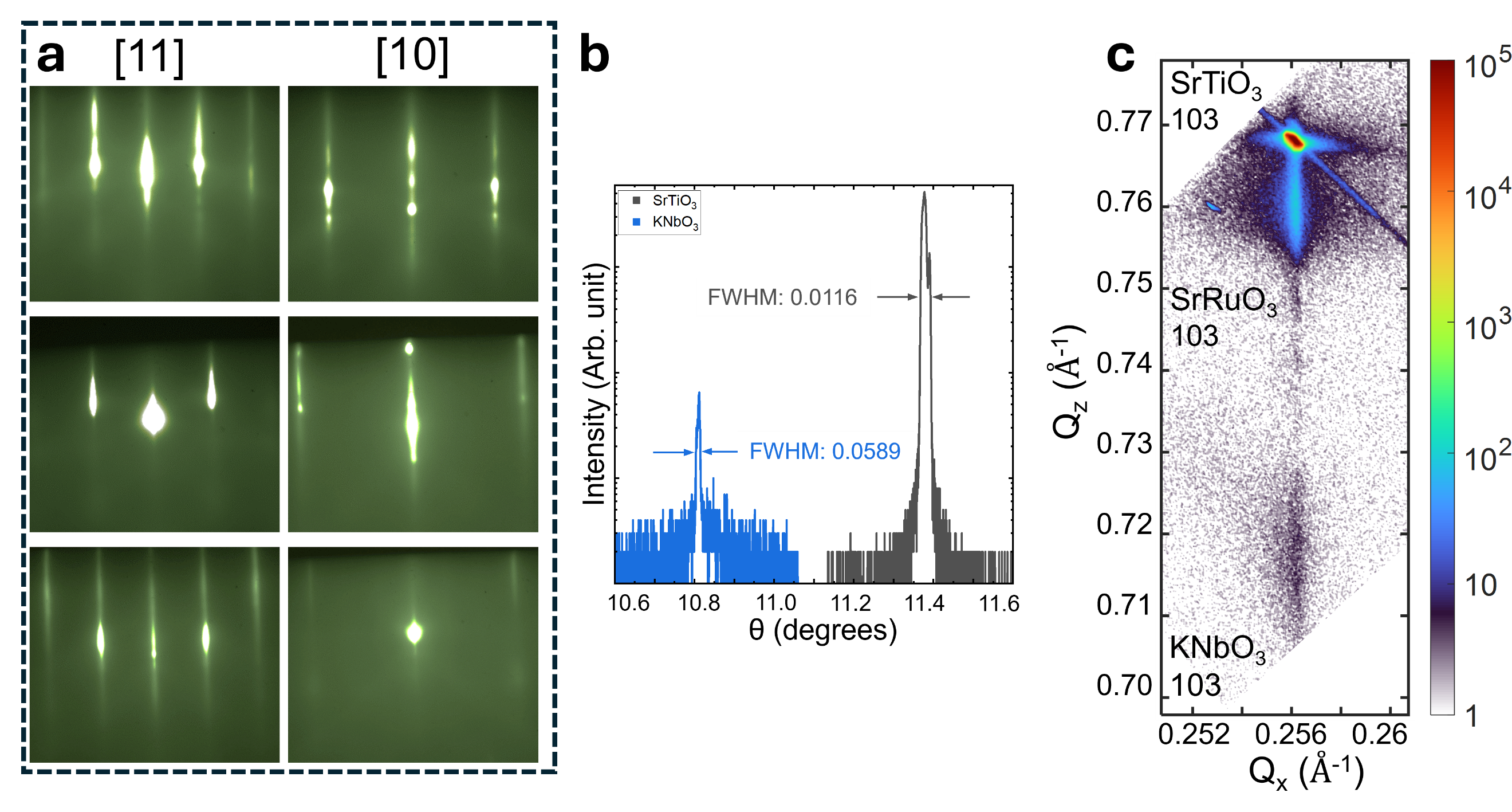


**Figure S18**: Structural characterization of a 19.8 nm thick KNbO_3_/SrRuO_3_/SrTiO_3_. (a) RHEED patterns of the bare SrTiO_3_ 001 substrate (top), SrRuO_3_ 001 bottom electrode (middle) and the KNbO_3_ film (bottom). (b) Overlaid 001 SrTiO_3_ and 001 KNbO_3_ peaks showing comparable FWHM values. (c) RSM of the KNbO_3_/SrRuO_3_/SrTiO_3_ heterostructure indicating that both the bottom electrode and the KNbO_3_ film are commensurately strained to the substrate.


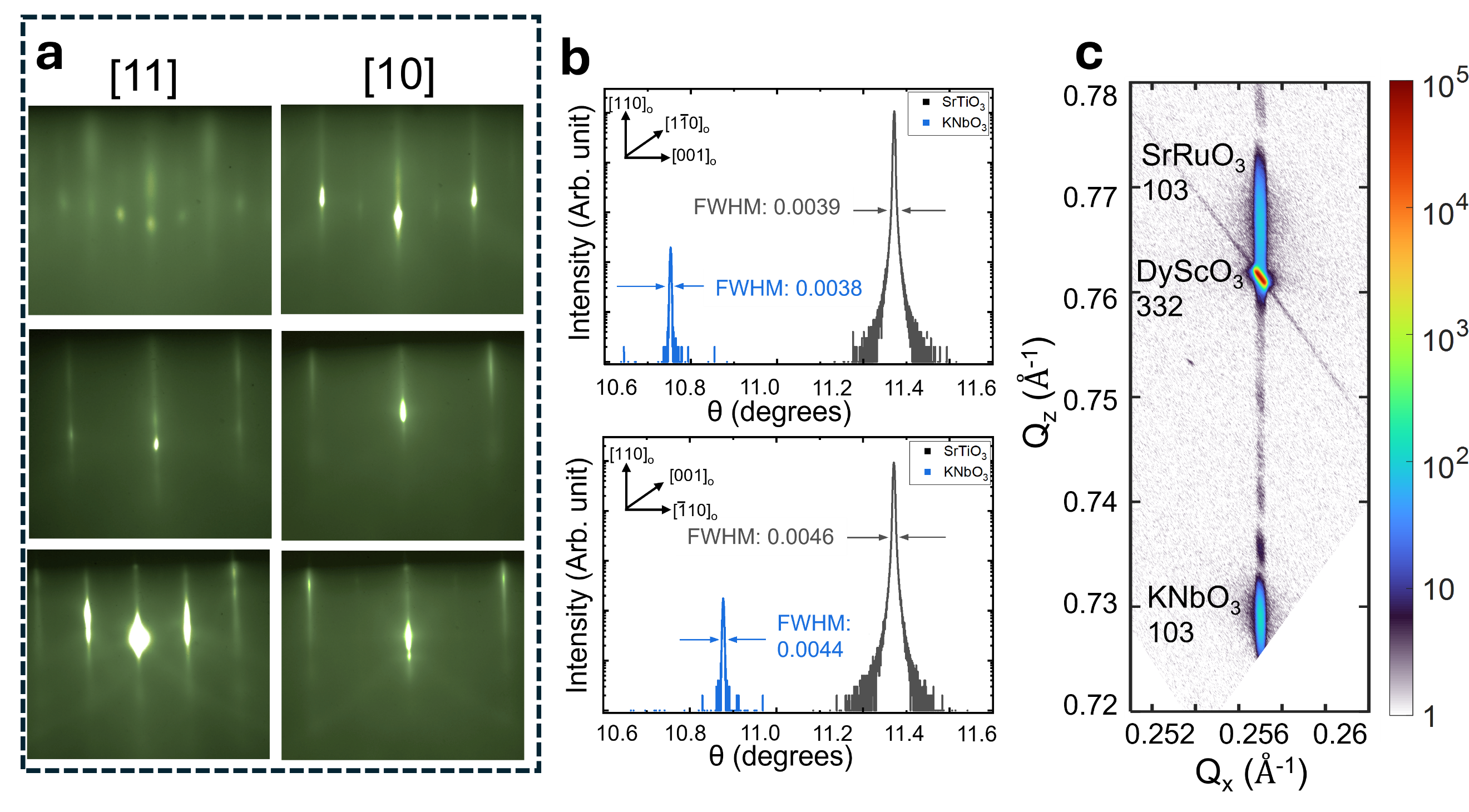


**Figure S19**: 21.8 nm-thick KNbO_3_/SrRuO_3_/DyScO_3_ (a) RHEED patterns of the bare SrTiO_3_ 001 substrate (top), SrRuO_3_ 001 bottom electrode (middle) and the KNbO_3_ film (bottom). (b) Overlaid 110 DyScO_3_ and 001 KNbO_3_ peaks showing comparable FWHM values. (c) RSM of the KNbO_3_/SrRuO_3_/DyScO_3_ heterostructure indicating that both the bottom electrode and the KNbO_3_ film are commensurately strained to the substrate


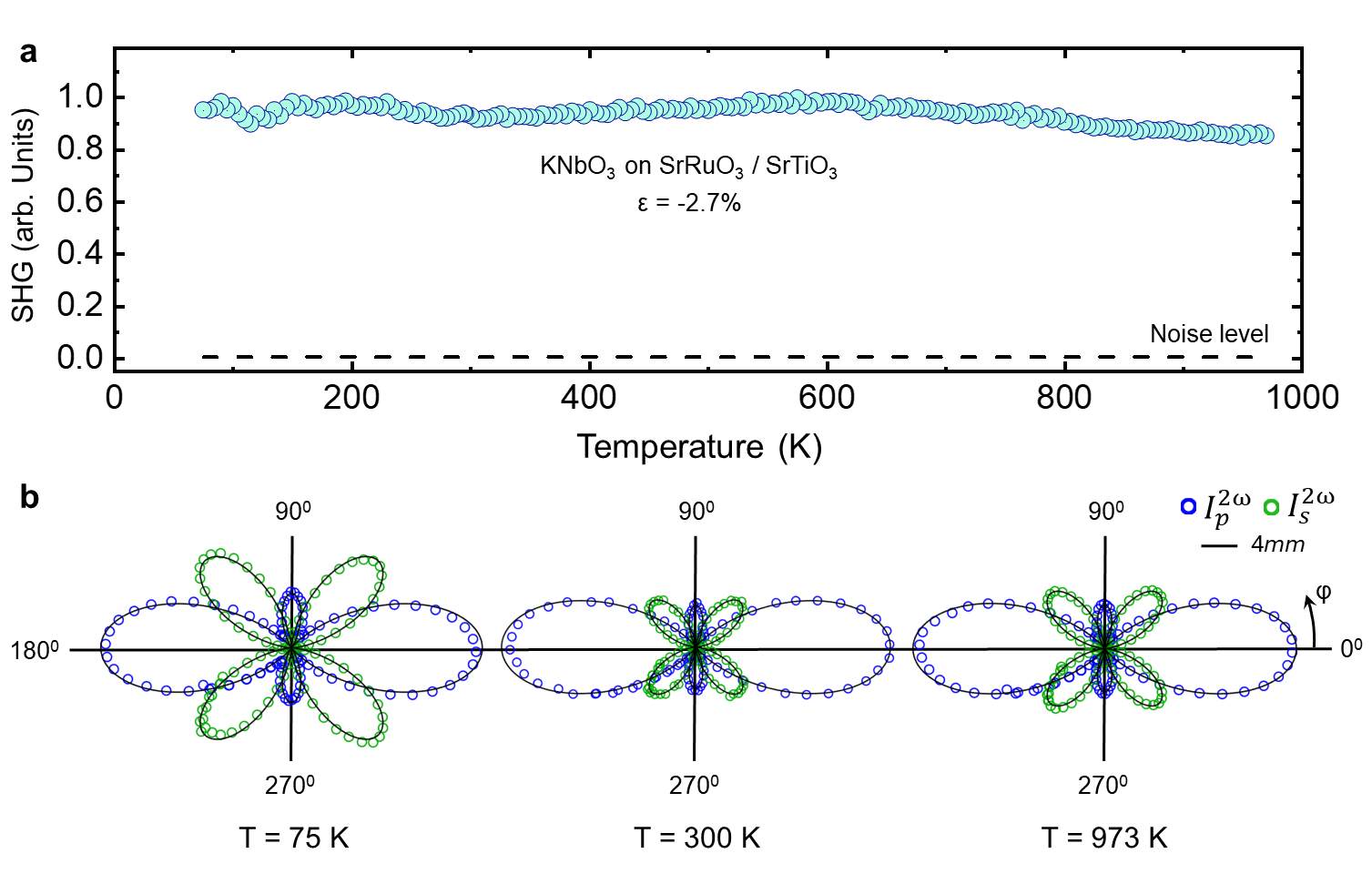


**Figure S20: a)** Temperature dependent SHG intensity of KNbO_3_/SrRuO_3_/SrTiO_3_ sample showing absence of any structural phase transitions and *T*_c_ pushed above 975 K consistent with phase-field simulations and SHG measurements on KNbO_3_ films without bottom electrode. **b)** SHG polarimetry done at 75 K, 300 K and 973 K fitted to tetragonal 4*mm* with out-of-plane polarization direction

**Note 13: Electrical characterization of polarization switching in strained KNbO_3_**

**Figure S21a** shows the PFM switching spectroscopy measured on the free surface of KNbO_3_/SrRuO_3_/DyScO_3_ suggesting a strong upward built-in field. The relaxation measurement in **Figure S21b** suggests that the downward state can be maintained for only ~10 ms, making polarization preferentially in the upward single domain state for the as-grown films. **Figure S21c** is the PFM switching spectroscopy on the free surface of KNbO_3_/SrRuO_3_/SrTiO_3_ with a strong upward built-in field leading to both coercive voltages positive, an indication that only upward polarization can be maintained.

**Figure S22a** shows the raw PUND data of the applied voltage and the recorded current from the capacitors, where high leakage is observed for the positive voltages, while much lower leakage is recorded for the negative voltages and the switching current peak can be extracted. **Figure S22b** displays the extracted switching peaks from the PUND measurements with various voltage pulse amplitudes from 3 V to 5.5 V. The calculated 2*P*_r_ values are shown in **Figure S22c**, which show a linear increase over the pulse amplitudes suggesting that there is an additional leakage contribution during the polarization switching process which is not subtracted from the PUND non-switching contributions (capacitive current and steady state leakage). Additional testing shows that 3 V of pulse amplitude leads to incomplete switching, so that 4 V is used for the estimation of the upper limit of the remanent polarization value. **Figure** **S22d** shows a PFM measurement for the polarization relaxation from the downward to upward state after a pulse of 3 V to set the initial state into downward. The PFM phase relaxed from ~0° (corresponding to downward state) to ~180° (corresponding to upward state) in about 10 s. **Figure** **S22e** shows an *I*-*V* measurement after the capacitor is set to the downward state with 4 V pulse. The first cycle starting with negative voltages shows no switching, suggesting that polarization is relaxed back to upward with a ~30 s delay time. **Figure** **S22f** is an AFM topography image of the 5×5 µm^2^ top electrode for the capacitors under investigation.

.


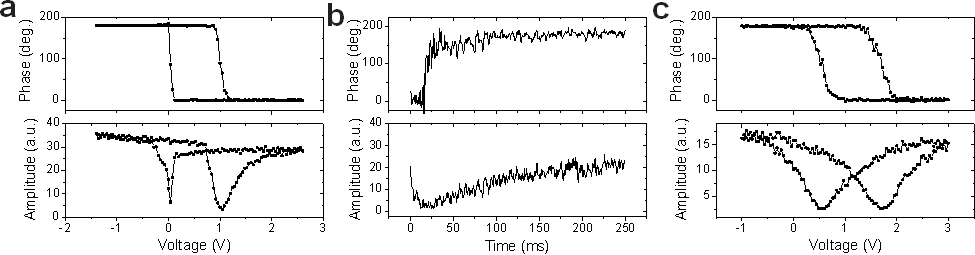


**Figure S21**. PFM switching spectroscopy and relaxation measurements on the free surface of KNbO_3_ thin films. (a) PFM switching spectroscopy on the 21.8 nm-thick KNbO_3_/SrRuO_3_/DyScO_3_ shows a strong upward built-in field that makes polarization down very unstable. (b) PFM measurement of the polarization relaxation of the downward state after a 4 V pulse shows polarization downward relaxes back to upward in 10~20 ms. (c) PFM switching spectroscopy on the 19.8 nm thick KNbO_3_/SrRuO_3_/SrTiO_3_ shows a strong upward built-in field leading to both coercive voltages positive.


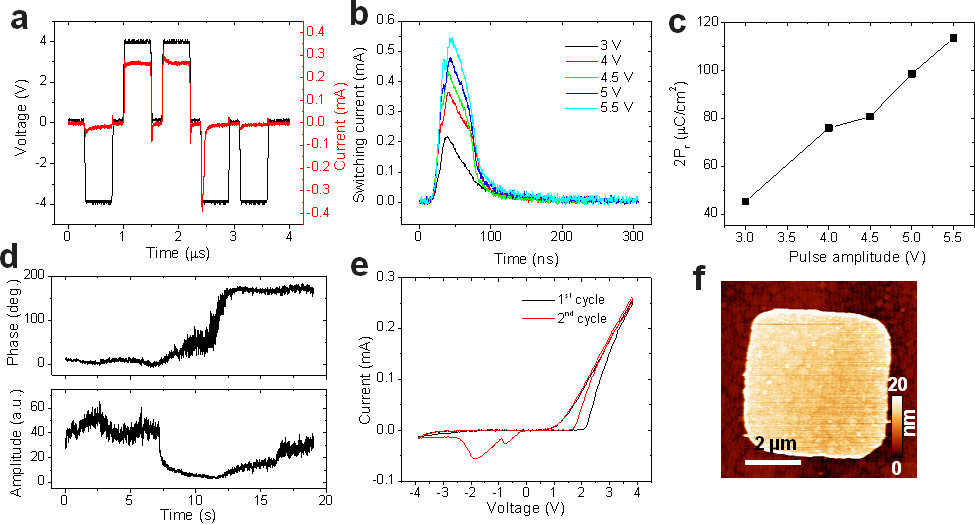


**Figure S22**. Electrical and PFM measurements on the Pt/KNbO_3_/SrRuO_3_/DyScO_3_ capacitors. **(a)** Raw PUND data of the applied voltage and the recorded current from the capacitor shows high leakage current for the positive voltage, while much lower leakage is recorded on the negative voltages enabling the extraction of the switching current peak. **(b)** The switching current peaks from the PUND measurements at various voltage pulse amplitudes from 3 V to 5.5 V. **(c)** The calculated switching charges (2*P*_r_) from the PUND measurements in (b) shows a linear increase with the pulse amplitude, which indicates a dynamic leakage during the polarization switching process. **(d)** PFM measurement of the relaxation of the downward polarization state after a pulse of 3 V, 10 ms, which relaxes to the upward state in about 10 s. **(e)** *I*-*V* measurement ~30 s after application of a 4 V pulse to set the initial polarization state downward, with a sweeping voltage from 0 V → – 4 V → +4 V → 0 V and repeat again. The first cycle on the negative side shows no polarization switching, suggesting that polarization has been relaxed to the upward state during the 30 s delay, while the second cycle shows clear switching peaks on the negative side. **(f)** An AFM topography image of the 5×5 µm^2^ top electrode.


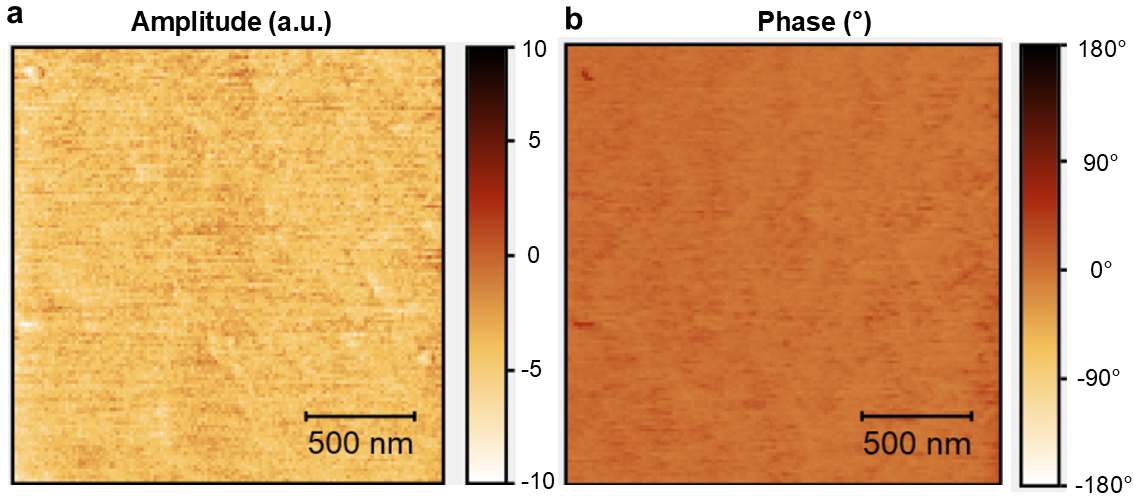


**Figure S23.** Room temperature piezoresponse force microscopy (PFM) maps across 2×2 µm^2^ region on KNbO_3_/SrRuO_3_/DyScO_3_ film showing no amplitude (**a**) and phase contrast (**b**) confirming the absence of 180° tetragonal domains.

**Note 14: Dielectric loss and electrical leakage characterization of KNbO_3_ films**

To investigate the dominant conduction mechanisms responsible for the rise in leakage current in KNbO₃ films, leakage current measurements were performed on 21.8 nm thick KNbO_3_ films grown on DyScO_3_ with a 15 nm thick SrRuO_3_ bottom electrode, using a 4140 Pico-Ampere Meter/DC Voltage Source (Hewlett Packard). 100 nm thick Pt electrodes, which were lithographically patterned into 50 μm x 50 μm squares, were used as the top electrode.


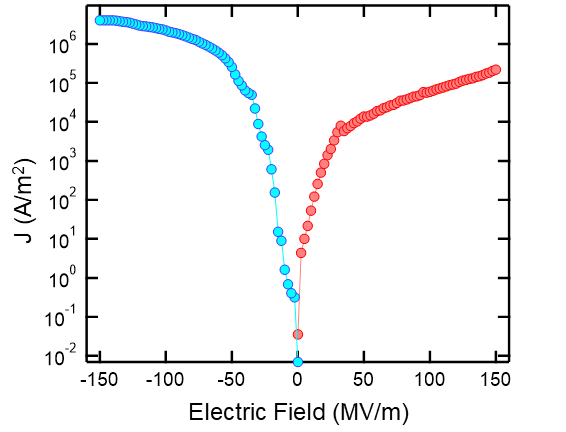


**Figure S24:** Polarity-dependent leakage current density (J) with increasing applied electric field. The blue curve corresponds to the field-down configuration (bottom electrode negatively biased). The red curve corresponds to the field-up configuration (top electrode negative biased)

A noticeable difference in the leakage current behavior was observed for opposite polarities **(Figure S24).** The leakage current increases more significantly with applied voltage when the bottom electrode is negatively biased compared to the top electrode (field-down). Conversely, the current levels are lower when the top electrode is negatively biased (field-up). This is consistent with PFM measurements of switching current shown in **Main Text, Figure 4b,** and suggests a significant contribution of an *interface-controlled mechanism* in the observed asymmetry of the leakage current.


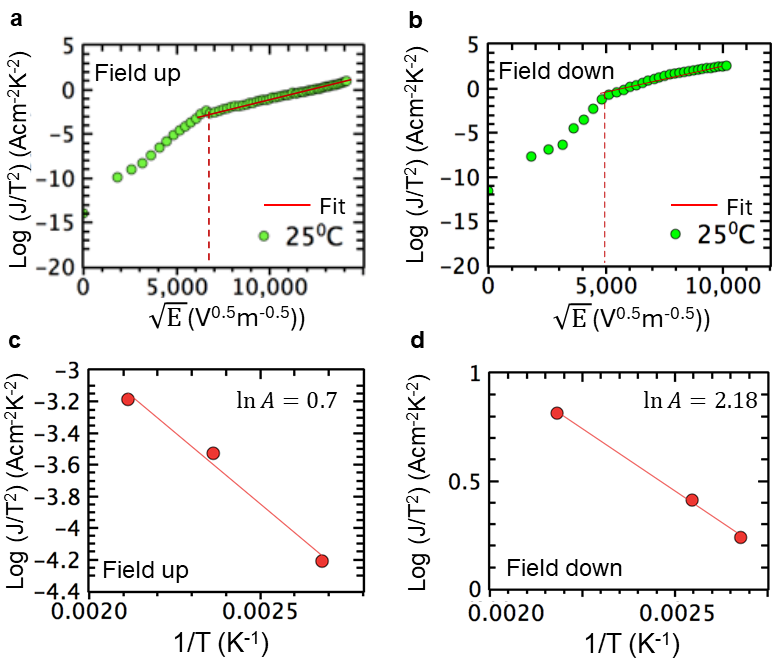


**Figure S25:** Current density vs electric field (ln(J/T²) versus E¹/²) fitted to Equation 1 showing the onset of Schottky emission for field-up **(a)** and field-down **(b)** configurations. Estimation of Richardson constant ($A$) from intercept of the ln (J/T^2^) versus 1/T at a given field for field-up **(c)** and field-down **(d)** configurations.

To further investigate the leakage characteristics, the temperature dependent I-V data was fitted to equations representing various interface-controlled conduction mechanisms. The current density-electric field (*J-E*) characteristics in KNbO₃ display a good linearity consistent with Schottky emission (ln(J/T²) versus E¹/²) for both polarities **(Figure S25a - b)**. The optical dielectric permittivity, $\varepsilon_{r}$, was extracted from the slope of the linear fits, and the refractive index, n $=\sqrt{\varepsilon_{r}}$​​, was calculated. The refractive index derived from the Schottky plots ranged from 2.2 to 2.9, which is in good agreement with the refractive index of KNbO_3_ (n ≈ 2.25) ^[21]^. This suggests that interface dominated Schottky emission to be one of the dominant contributors of the leakage in these KNbO₃ films.

To estimate the Schottky barrier height, the Richardson constant, $A$, was first determined from the intercept of the ln (J/T^2^) versus 1/T at a given field **(Figure S25c - d)**. Then, the Schottky barrier height (Φ_B_) was extracted from the intercept of ln (J/T^2^) vs. E^1/2^ plot using the following equation:

$\ln\left( \frac{J_{Sch}}{T^{2}} \right)=lnA-\frac{q\Phi_{B}^{Sch}}{k_{B}T}+\frac{q\sqrt{\frac{qE}{4\varepsilon_{0}\varepsilon_{\infty}\pi}}}{k_{B}T}$ Equation 1

where A is the Richardson constant, ε_∞_ is the high frequency permittivity, $\Phi_{B}^{Sch}$ is the barrier height, and *q* is the charge per carrier. Φ_B_ is estimated to be 0.4±0.04 eV for the top Pt/KNbO_3_ interface and 0.25±0.03 eV for the bottom SrRuO_3_/KNbO_3_ interface. The difference in Φ_B_ is likely due to the difference in work function of top Pt (5.6 eV)^[22]^ and bottom SrRuO_3_ (5.2 eV)^[23]^ electrodes. The higher work function of Pt results in a higher Φ_B_, which leads to lower leakage current levels under field-up conditions and contributes to the polarity-dependent leakage current behavior observed in KNbO₃ films. A schematic representation of the band bending in KNbO_3_ at both interfaces is shown in **Figure S26**.


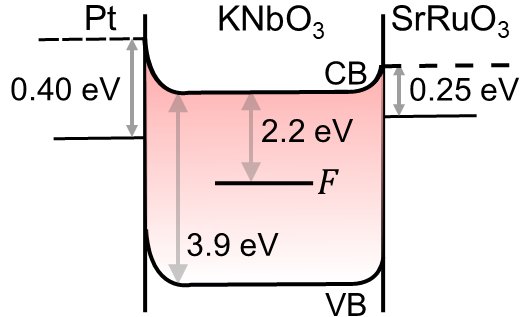


**Figure S26:** Schematic band diagram of KNbO_3_ showing the measured Schottky barrier heights at both Pt-KNbO_3_ and SrRuO_3_-KNbO_3_ interfaces and known *F*-center (Oxygen-vacancy which traps electrons) energy level in KNbO_3_ ^[24,25]^. The *F*-center energy level in KNbO_3_ is deep due to its molecular orbital being primarily contributed by the two nearest Nb atoms from the oxygen vacancy ^[24]^.

To further explore the charge transport mechanisms in the KNbO_3_ film, temperature dependent modulus spectroscopy measurements were performed using a Solartron 1260 Impedance analyzer with a l00 mV AC amplitude, over a frequency range from 1 MHz to 0.01 Hz. **Figure S27** shows the frequency dependence of the imaginary part of the electric modulus (*M''*(*f*)) of the measured the KNbO₃ film at temperatures ranging from 30°C to 200°C.

*M''*(*f*) exhibits a maximum at the relaxation frequency, *f(*r), which can indicate the volume fraction of material with similar conductivity. The relaxation frequency *f*(r) of the modulus peak is expressed as:

$f\left( r \right)=\frac{1}{2\pi RC}=\frac{\sigma}{2\pi\epsilon_{0}\epsilon_{r}}$ Equation 2

where *σ is* the conductivity, *ε*_r_ is the relative permittivity, C is capacitance, and R is resistance.

The temperature dependence of the modulus peak was analyzed to determine the origin of the charge transport mechanism (**Figure S27a**). By fitting the conductivity calculated through Equation 2 to the Arrhenius equation, the activation energy related to dielectric relaxation associated with the observed modulus peak is calculated to be 0.45 ± 0.04 eV (**Figure S27b**).

The exact mechanism responsible for such relaxation behavior is unknown. We speculate that this relaxation process is related to polaron hopping mechanism which has previously been observed in KNbO_3_ single crystals ^[26,27]^. Oxygen vacancies are one of the main sources of point defects in KNbO₃ which acts as electron donors. These electrons can be bound to Nb^5+^ ion cation forming a polaron. Hopping of such polarons between symmetry equivalent sites results in increased conductivity.


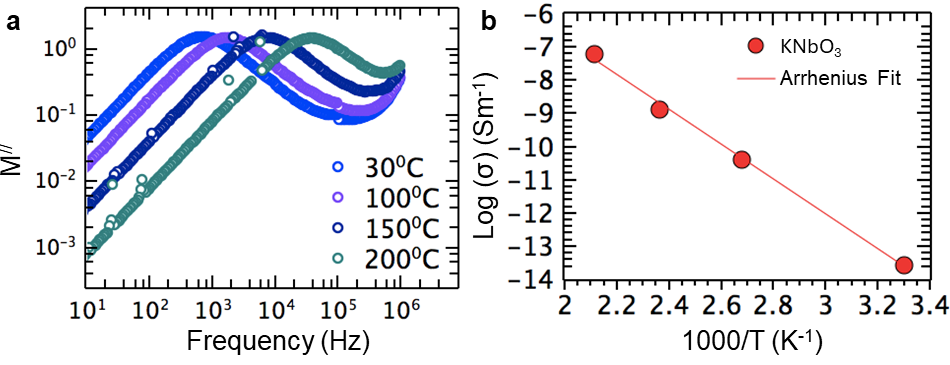


**Figure S27:** **(a)** Imaginary part of electric modulus (*M''*) as a function of frequency showing a dielectric relaxation peak moving to higher frequencies with increasing temperature. **(b)** *σ* vs T curve (Log *σ* vs 1000/T) corresponding to the observed dielectric relaxation peak fitted to the Arrhenius equation.

**Origin of defects in KNbO_3_**:

Potassium vacancies and oxygen vacancies are the primary point defects in KNbO_3_. Due to the high K vapor pressure, K vacancies form on high temperature exposure of KNbO_3_, including during growth. Subsequently, this leads to oxygen vacancies, in order to maintain charge neutrality, as can be described in Kroger-Vink notation as follows:

$2K_{K}+O_{O}\to2V_{K}^{'}+V_{O}^{\cdot}+K_{2}O(\uparrow)$ (Equation 3)

Since these oxygen vacancies are necessary for charge neutrality, they are thermodynamically stable and cannot be removed via annealing. Localized hopping of such singly ionized oxygen vacancies can lead to dielectric relaxation ^[28]^. A secondary pathway of forming oxygen vacancies is due to oxygen leaving lattice positions; these defects serve as electron donor defects, which can be expressed in Kroger-Vink notation as follows:

${2O}_{O}\to+V_{O}^{**}+2e^{'}+O_{2}(\uparrow)$ (Equation 4)

These electrons could be weakly bound to Nb^5+^ cations, forming dipoles giving rise to dielectric relaxation mechanisms in KNbO_3_ ^[28,29]^. Oxygen vacancies in KNbO_3_ can also serve as electron trapping sites (*F*-centre) which produces deep energy levels, ~ 2.2 eV below the conduction band ^[24]^ in KNbO_3_ (**Figure S26**). A high concentration of oxygen vacancies in KNbO₃ films can also modulate the potential barrier height via Fermi level pinning, accelerating charge injection under DC electric fields. Consequently, the distribution of oxygen vacancies throughout the thickness of KNbO₃ films can contribute to asymmetric leakage current characteristics and polarity-dependent lifetimes. Furthermore, oxygen vacancies can contribute to both electronic and ionic conductivity in KNbO₃ films ^[28]^.

Finally, migration of K^+^ ions and oxygen vacancies, having activation energies of 0.6 eV and 1.2 eV, respectively, are another source of conduction (especially at high temperatures) which has been observed in KNbO_3_ single crystals ^[30]^. The formation of conductive domain walls and pinholes across a thin film are also possible contributors to leakage in KNbO_3_ thin films.

Further investigations are necessary to understand the relative influence of different defect mechanisms involved in high leakage current levels in KNbO_3_ films.

**Improving leakage characteristics of KNbO_3_**:

All said, the primary source of leakage and slower switching dynamics in KNbO_3_ is understood to be due to oxygen vacancies formed either due to K vacancy formation or O leaving lattice positions during material processing which contribute to the dielectric leakage properties of KNbO_3_. A possible method to eradicate this is by growing KNbO_3_/SrTiO_3_ superlattices. Superlattices on substrates on SrTiO_3_ or DyScO_3_ substrates, similar to the well-studied PbTiO_3_/SrTiO_3_ superlattice system^[31]^, will allow the growth of thicker films while maintaining coherent strain in KNbO_3_. This will help eradicate mechanical sources of leakage such as pinhole formation in films. More importantly, in superlattice heterostructures, KNbO_3_ can be capped with a SrTiO_3_ layer, which will reduce the loss of K from the film. This in turn should significantly decrease oxygen vacancy concentrations in the films leading to improved dielectric loss characteristics. Finally, doping the KNbO_3_ with a small amount of electron acceptors such as Ba^2+^ and Sr^2+^ can as also potentially reduce leakage.

**References**

[1] L. Liang, Y. L. Li, L.-Q. Chen, S. Y. Hu, G.-H. Lu, *J Appl Phys* **2009**, *106*, DOI 10.1063/1.3260242.

[2] A. Nazeri-Eshghi, A. X. Kuang, J. D. Mackenzie, *J Mater Sci* **1990**, *25*, 3333.

[3] S. Triebwasser, *Physical Review* **1956**, *101*, 993.

[4] G. Shirane, H. Danner, A. Pavlovic, R. Pepinsky, *Physical Review* **1954**, *93*, 672.

[5] G. Sheng, Y. L. Li, J. X. Zhang, S. Choudhury, Q. X. Jia, V. Gopalan, D. G. Schlom, Z. K. Liu, L. Q. Chen, *Appl Phys Lett* **2010**, *96*, DOI 10.1063/1.3442915.

[6] M. J. Haun, E. Furman, S. J. Jang, H. A. McKinstry, L. E. Cross, *J Appl Phys* **1987**, *62*, 3331.

[7] Y. L. Li, L. E. Cross, L. Q. Chen, *J Appl Phys* **2005**, *98*, DOI 10.1063/1.2042528.

[8] H. Uwe, T. Sakudo, *J Physical Soc Japan* **1975**, *38*, 183.

[9] M. J. Weber, *Handbook of Optical Materials*, CRC Press, **2018**.

[10] L. Chen, *Journal of the American Ceramic Society* **2008**, *91*, 1835.

[11] B. Wang, L.-Q. Chen, *Acta Mater* **2024**, *274*, 120025.

[12] B. Wang, H.-N. Chen, J.-J. Wang, L.-Q. Chen, *Appl Phys Lett* **2019**, *115*, DOI 10.1063/1.5116910.

[13] L. F. Wan, T. Nishimatsu, S. P. Beckman, *J Appl Phys* **2012**, *111*, DOI 10.1063/1.4712052.

[14] R. Uecker, B. Velickov, D. Klimm, R. Bertram, M. Bernhagen, M. Rabe, M. Albrecht, R. Fornari, D. G. Schlom, *J Cryst Growth* **2008**, *310*, 2649.

[15] W. Zheng, Y. Tang, C. Jia, Z. Liu, Z. Zhang, K. Zhao, *J Mater Chem A Mater* **2024**, *12*, 11378.

[16] A. Winiarski, T. Neumann, B. Mayer, G. Borstel, M. Neumann, *physica status solidi (b)* **1994**, *183*, 475.

[17] T. Zhang, K. Zhao, J. Yu, J. Jin, Y. Qi, H. Li, X. Hou, G. Liu, *Nanoscale* **2013**, *5*, 8375.

[18] V. Gopalan, R. Raj, *Appl Phys Lett* **1996**, *68*, 1323.

[19] V. Gopalan, R. Raj, *J Appl Phys* **1997**, *81*, 865.

[20] R. Zu, B. Wang, J. He, L. Weber, A. Saha, L.-Q. Chen, V. Gopalan, *NPJ Comput Mater* **2024**, *10*, 64.

[21] Y. Shiozaki, E. Nakamura, T. Mitsui, Eds., *Oxides*, Springer-Verlag, Berlin/Heidelberg, **2002**.

[22] B. Ofuonye, J. Lee, M. Yan, C. Sun, J.-M. Zuo, I. Adesida, *Semicond Sci Technol* **2014**, *29*, 095005.

[23] V. Sampath Kumar, M. K. Niranjan, *J Appl Phys* **2014**, *115*, DOI 10.1063/1.4872466.

[24] R. I. Eglitis, E. A. Kotomin, A. V. Postnikov, N. E. Christensen, M. A. Korotin, G. Borstel, *Ferroelectrics* **1999**, *229*, 69.

[25] R. I. Eglitis, N. E. Christensen, E. A. Kotomin, A. V. Postnikov, G. Borstel, *Phys Rev B* **1997**, *56*, 8599.

[26] J. Handerek, R. Manka, A. Aleksandrowicz, J. Szatanek, *Ferroelectrics* **1978**, *22*, 735.

[27] S. Torbrügge, M. Imlau, B. Schoke, C. Merschjann, O. F. Schirmer, S. Vernay, A. Gross, V. Wesemann, D. Rytz, *Phys Rev B* **2008**, *78*, 125112.

[28] G. Singh, V. S. Tiwari, P. K. Gupta, *J Appl Phys* **2010**, *107*, DOI 10.1063/1.3309745.

[29] Y. Wu, M. J. Forbess, S. Seraji, S. J. Limmer, T. P. Chou, G. Cao, *J Appl Phys* **2001**, *89*, 5647.

[30] R. I. Eglitis, E. A. Kotomin, G. Borstel, *physica status solidi (c)* **2005**, *2*, 113.

[31] A. K. Yadav, C. T. Nelson, S. L. Hsu, Z. Hong, J. D. Clarkson, C. M. Schlepütz, A. R. Damodaran, P. Shafer, E. Arenholz, L. R. Dedon, D. Chen, A. Vishwanath, A. M. Minor, L. Q. Chen, J. F. Scott, L. W. Martin, R. Ramesh, *Nature* **2016**, *530*, 198.
